# Neonatal liver niches program T cell tolerance

**DOI:** 10.64898/2026.01.13.698576

**Published:** 2026-01-13

**Authors:** Eva-Lena Stange, Trong-Hieu Nguyen, Dustyn Mendoza, Aiara Lobo Gomes, Marlene Sophia Kohlhepp, Jonas Pes, Shahed Al Bounny, Julian Brueck, Yunus Cetiner, Urs Moerbe, Milas Ugur, Cristina Kalbermatter, Jarrett Lopez-Scarim, Aline Dupont, Susan A. V. Jennings, Julia Heckmann, Aaron Silva-Sanchez, Solveig Runge, Christoph Kuppe, Oliver Pabst, Thomas Clavel, Dorothee Viemann, Stephan P. Rosshart, Vuk Cerovic, Stephanie C. Ganal-Vonarburg, Adrien Guillot, Eva Billerbeck, Mathias W. Hornef, Natalia Torow

**Affiliations:** 1 Institute of Medical Microbiology, University Hospital RWTH Aachen; Aachen, Germany.; 2 Institute of Molecular Medicine, University Hospital RWTH Aachen; Aachen, Germany.; 3 Department of Medicine, Division of Hepatology, and Department of Microbiology & Immunology; Albert Einstein College of Medicine, New York, USA.; 4 Charité-Universitätsmedizin Berlin, Department of Hepatology & Gastroenterology; Campus Virchow-Klinikum and Campus Charité Mitte, Berlin, Germany.; 5 Department of Immunology & Microbiology, LEO Foundation Skin Immunology Research Center, University of Copenhagen; Copenhagen, Denmark.; 6 Institute of Systems Immunology, Julius-Maximilians-Universität Würzburg, Würzburg, Germany; 7 Department of Visceral Surgery and Medicine, Inselspital, Bern University Hospital, University of Bern, Switzerland; 8 Department for Biomedical Research, University of Bern; Bern, Switzerland.; 9 Functional Microbiome Research Group, Institute of Medical Microbiology, University Hospital RWTH Aachen; Aachen, Germany.; 10 Department of Pediatrics, University Hospital Würzburg; Würzburg, Germany.; 11 Department of Medicine, Division of Clinical Immunology and Rheumatology, The University of Alabama at Birmingham Heersink School of Medicine; Birmingham, AL 35294, USA.; 12 Department of Microbiome Research, University Hospital Erlangen, Friedrich-Alexander-Universität Erlangen-Nürnberg (FAU); Erlangen, Germany.; 13 Department of Medicine 1, University Hospital Erlangen, Friedrich-Alexander-Universität Erlangen-Nürnberg (FAU); Erlangen Germany.; 14 Division of Nephrology and Clinical Immunology,Medical Faculty, RWTH Aachen University; Aachen, Germany.; 15 Center for Computational Life Sciences, RWTH Aachen University; Aachen, Germany.; 16 Helmholtz Centre for Infection Research (HZI); Braunschweig, Germany.

## Abstract

After birth, the immune system must learn to tolerate a rapidly changing milieu of commensals and self while remaining ready for pathogens. Here we characterize the neonatal liver as a central hub in this process: In postnatal week 1–2, the liver hosts a developmentally encoded, microbiota-independent expansion of regulatory T cells (Tregs) that coexists with microbiota-tuned conventional wave of activated CD4^+^ T cells (Tconvs). Mechanistically, the Treg expansion is governed by MHCII-mediated antigen presentation by CCR7+ cDC1s, which establish tolerogenic DC:T cell clusters in the liver parenchyma, allowing for local expansion and control via PD-L1 checkpoints that selectively increase Tregs without unleashing Tconvs. Importantly, this transient, neonatal program predisposes hepatotropic viral infections to progress toward chronic disease but also protects the adult liver from steatotic disease. These data position the neonatal liver as a unique site of early life T-cell education with timing-sensitive implications for early-life interventions.

## Introduction

Birth marks a distinct phase for adaptive immunity in the murine host: peripheral tissues are rapidly seeded by thymus-derived recent thymic emigrants (RTEs), and the early peripheral T cell pool is strongly enriched for cells that are more self-reactive and less stringently tuned than adult naïve T cells (1–4). At the same time, microbial colonization takes hold at barrier surfaces (5, 6). Therefore, the neonatal immune system must enforce peripheral self-tolerance and, in parallel, establish tolerance to newly encountered commensals.

Consequently, early life immunity is characterized by regulatory T cell (Treg) waves, although the timing and mechanism of induction differ by tissue. In the intestine, T cells remain naïve and have restricted tissue access until weaning, when a major dietary transition arrives with solid food, and an intestine-specific, microbiota- and dietary antigen-dependent Treg burst has been described (7–9). This gut-specific Treg burst is induced by the maturation and development of intestinal antigen uptake mechanisms at weaning, including goblet cell–associated passages and M-cell–mediated transcytosis (10–13). Although early-life Treg expansion has been most intensively studied in the gut, pre-weaning Treg waves have also been documented in lung and skin, where transient, microbiota-dependent expansions curb inflammatory priming (14–16). Together, these observations raise a broader question: where and how is tolerance enforced prior to the maturation of classical intestinal sampling?

In the liver, a pre-weaning Treg wave has been noted previously (17, 18), implicating the liver as a key tolerogenic environment in early life. Further, in the adult liver, antigen presentation and coinhibitory pathways bias adaptive immunity towards deletion, anergy, or Treg induction (19–23). Moreover, the liver can also organize structured immune microenvironments in inflammatory settings, underscoring its capacity to support local immune organization (19, 21, 24). However, the mechanisms of induction, antigen presentation and microbiota dependence as well as the functional relevance of the early-life hepatic Treg wave remain largely unclear. Elucidating these tolerogenic molecular pathways represents an urgent need for the rational design of effective vaccines and therapies targeting neonatal infections.

Here, we define an early-life immune architecture in which the neonatal liver functions as a transient, tolerogenic CD4^+^ T cell niche. We demonstrate that the neonatal hepatic Treg wave represents a developmentally programmed, microbiota-independent, locally controlled Treg expansion that occurs concurrently with a microbiota-mediated Tconv activation. Crucially, we identify CCR7^+^ type 1 conventional dendritic cells (cDC1s) as the dominant antigen presenting cell (APC) population that anchors Treg- and Tconv-containing clusters within the neonatal liver parenchyma. Furthermore, we show the key role of cDC1-associated PD-L1 signaling in shaping the balance between Treg accumulation and Tconv activation, providing a mechanism by which the neonatal liver calibrates responses to portal-derived microbial constituents. Finally, we link this early-life tolerogenic set-point to delayed control of pediatric hepatotropic virus infection and increased resistance to metabolic-induced liver inflammation in the adult host.

### A developmentally-programmed postnatal hepatic Treg wave constrains microbiota-dependent Tconv expansion and activation

**Figure 1:**
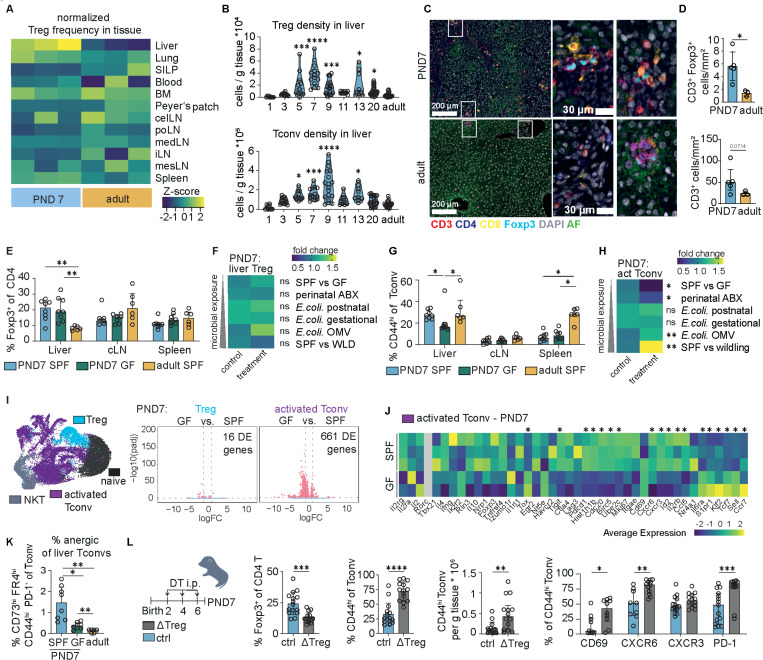
A transient Treg peak restrains microbiota-tuned Tconv activation across hepatic clusters (A) Row z-score normalized data % of Treg within the CD4 T cell compartment in liver, lung, small intestinal lamina propria (siLP), blood, bone marrow (BM), Peyer’s Patch, celiac lymph node (cLN), portal lymph node (PLN), mediastinal lymph node (medLN), inguinal lymph node (iLN) and mesenteric lymph node (mLN) and spleen in adult and 7-day old neonatal mice. Flow cytometry, 3 mice per group, Representative data from 1–5 experiments. (B) Foxp3^+^ CD4 T cells (Treg) and Foxp3^−^ CD4T cells (Tconv) per g liver tissue over the pre-weaning period. Violin plots of 6–20 mice per timepoint from 2–6 independent experiments. Kruskal Wallis test + post hoc Dunn’s comparison of each time point to the adult group. (C) Immunofluorescence of T cell markers (CD3, CD4, CD8α, Foxp3) in neonatal (PND7) and adult liver tissue. (D) Automated full-slide quantification of in (c): CD3^+^ and Foxp3^+^ neonatal and adult liver tissue from 3–5 mice group. Welch t-tests. (E) % of Treg within CD4 T cell compartment in liver, celiac lymph node (cLN) and spleen (Spl) of neonatal (PND7) SPF and GF mice and adult SPF mice. 6–8 mice per group from two independent pooled experiments. Brown-Forsythe ANOVA + Dunnett’s multiple comparison test (liver, cLN) or One Way ANOVA + post hoc Holm-Sidak’s multiple test (Spl). (F) Heatmap showing the mean fold change of % Treg of liver CD4 T cells normalised to the median of the respective control group (untreated/mock-treated SPF mice for comparison to GF, AVNM, OMV and WLD; untreated GF mice for GC and PC) of the different microbial colonization experiments shown in Ext. Data 2a. Asterisks represent the results of the statistical tests of the % of Treg data from Ext.data 2a-e (G) % of CD44^hi^ Tconv within CD4 T cell compartment in liver, celiac lymph node (CLN) and spleen (Spl) of neonatal (PND7) SPF and GF mice and adult SPF mice. 6–8 mice per group from two independent pooled experiments. Kruskal Wallis + Dunn’s multiple comparison test (Liver, spleen) or One Way ANOVA + post hoc Holm-Sidak’s multiple test (cLN). (H) Heatmap showing the mean fold change of % CD44^hi^ liver Tconv in the liver normalized to the median of the respective control group (untreated/mock-treated SPF mice for comparison to GF, AVNM, OMV and WLD; untreated GF mice for GC and PC) of the different microbial colonisation experiments shown in Ext. Data 2a. Asterisks represent the results of the statistical tests of the %CD44^hi^ of Tconv data from Ext.data 2a-e. (I) UMAP of liver CD4 T cells from different neonatal and adult time-points of SPF and GF and volcano plots showing the significant differentially expressed DE genes between 7-day old SPF and GF mice. (J) Expression of a curated list of genes between activated liver Tconvs (purple cluster) in neonatal (PND7) SPF and germ-free mice normalized column expression. Asterisks indicates DE gene between PND7 SPF and PND7 GF mice. (K) Frequency of anergic cells (defined as CD73^hi^ FR4^hi^ PD-1^+^ CD44^hi^ Tconvs) within Tconvs in 7-day-old SPF and GF liver as well as adult SPF liver measured by flow cytometry. 8 mice from 2 experiments. Brown-Forsythe ANOVA + Dunnett’s multiple comparison test. (L) Experimental layout of neonatal Treg depletion experiment; frequency of Treg and CD44hi Tconv in liver CD4 T cells and hepatic density of activated Tconvs measured by CD44 expression and expression of CD69, CXCR6, CXCR3 and PD-1 in CD44hi Tconv. 13–15 mice pooled from 4 independent experiments: Multiple Mann Whitney U tests, Welch t-test (% Treg of CD4 T cells, % CD44hi of Tconvs) or Mann Whitney U test (CD44hi Tconv density). All experiments containing flow cytometry were done with the compared groups processed on the same day within 1 experiment. Asterisks indicate significance levels: * < 0.05, ** < 0.01, *** < 0.001 **** < 0.0001.

A comparison of the CD4^+^ T cell compartment in early life and adulthood by flow cytometry revealed a notable enrichment of Tregs in the neonatal liver of specific-pathogen-free (SPF) mice, in marked contrast to other peripheral or lymphoid tissues (Fig. 1a) and in accordance with the previously reported “neonatal Treg wave” (17, 25). Likewise, the total hepatic CD4 and CD8 T cell density was higher in the neonatal liver but not spleen or small intestinal *lamina propria* (SILP) (Figs. S1A-B). To further explore the inductive mechanisms and functional implications of this early T cell/Treg wave, we performed a detailed kinetic analysis across the pre-weaning period (Extended data 1c-e). The hepatic CD4^+^ T cell compartment underwent a pronounced early-life expansion that peaked at postnatal day (PND) 7–9 (Fig. S1c). Within this wave, Tregs peaked at PND7, accounting for up to ~35% of hepatic CD4^+^ T cells (Fig. 1 b; Extended data 1d). Conventional CD4^+^ T cells (Tconv) expanded with slightly delayed kinetics, peaking at PND9 before both populations gradually declined toward adult set points (Fig. 1b). Liver CD8^+^ T cells showed a similar kinetic to CD4^+^ T cells, while non-classical T cell populations, such as TCRγδ^+^ T cells and NKT cells also showed substantial expansion in the pre-weaning period, albeit with distinct kinetics (Fig. S1e). Immunofluorescence imaging of hepatic tissue confirmed the increased density of T cells along with Tregs in neonatal livers and revealed clusters of co-localizing Tregs with other T cell subsets distributed throughout the liver parenchyma including periportal areas (Fig. S1c-d). In adult livers, T cell clusters were present at almost exclusively confined to periportal areas (Fig. S1f) and were less diverse in composition (Fig. 1c). Collectively, these data point to the neonatal liver as a key site of T cell immune activation and regulation in early life.

Previous reports have suggested that the neonatal Treg expansion in the liver is dependent on the intestinal microbiota(17, 25). To test this, we compared the hepatic T cell compartment of neonatal mice reared under SPF conditions to those of germ-free (GF) neonates. Surprisingly, the hepatic (and liver-draining lymph node) Treg frequency at PND7 remained unchanged (Fig. 1e). To further understand the dependence of neonatal T cells on stimuli from the microbiota and their timing, we analysed litters born after perinatal treatment of dams with broad-spectrum antibiotics, GF mice following “reversible colonization” with auxotrophic *E. coli* HA107 either during pregnancy or in the first week after birth, or neonatal SPF mice treated with *E. coli* outer membrane vesicles (OMVs) for 8h. Additionally, we compared neonatal SPF mice with “wildlings”, which harbour a more diverse microbiota. Strikingly, despite the highly variable strength and timing of microbial exposure, we observed no significant difference in the overall proportion of hepatic Tregs on PND7, demonstrating that the hepatic neonatal Treg wave develops independently of the host microbiota (Fig. 1f; Fig. S2a). In marked contrast, the proportion of neonatal activated CD44^hi^ Tconvs was significantly reduced in the liver but not the spleen or celiac lymph node of GF mice (Fig. 1g) as well as in livers of neonates from antibiotic-treated dams. Conversely, hepatic CD44^hi^ Tconvs showed a significant increase shortly after exposure of SPF neonates to bacterial OMVs as well as in wildlings (Fig. 1h; Figs. S2a-e). By adulthood, activated Tconv frequencies had equalized in germ-free mice, indicating that this distinct neonatal sensitivity to microbial exposure dissipates once the hepatic immune landscape matures (Fig. S2f). Collectively, these data show that, while the hepatic Treg wave is developmentally controlled, the Tconv compartment in the neonatal liver is sensitive to microbial cues.

In order to understand the identified changes in the liver immune landscape at this key period, we performed scRNAseq analysis of hepatic CD45^+^ leukocytes from neonatal and adult SPF and GF mice (Fig. S3). Transcriptional profiling underlined the dependence of hepatic neonatal Tconvs on microbiota signals as we observed an extensive reprogramming between GF and SPF CD44^hi^ Tconvs. In contrast, only minor differences were observed in the Treg compartment (Fig. 1i, Fig. S3h). Activated Tconvs in livers of GF neonates shifted towards a naïve/quiescent transcriptional signature (e.g., *Sell*, *Ccr7*, *Tcf7*) with lower expression of cell-cycle genes (*Mki67*, *Birc5*, *Ube2c*) relative to their SPF counterparts (Fig. 1j). Conversely, activated Tconvs from SPF neonates upregulated transcripts associated with T cell activation (*Il2rb, Id2, Cxcr3*; concomitant with downregulation of *Ccr7, Sell, Tcf7*). Notably however, SPF Tconvs also showed a parallel upregulation of genes encoding inhibitory/tolerance-associated receptors (*Tigit, Pdcd1*) and comprised a higher proportion of CD73^hi^ FR4^+^ anergic cells in flow cytometry (Fig. 1k).

These data fit a model in which the microbiota drives a controlled, tolerance-prone anergy state in Tconvs, seeding an anergic intermediate cell state that can differentiate into peripherally-induced (p)Tregs (18, 26). These observations, coupled with the temporal and spatial proximity of liver Tregs and Tconvs in early life, led us to hypothesise that the neonatal Treg wave functions to constrain the Tconv activation that is modulated by the microbiota. In line with this, Ova-specific OTII T cells adoptively transferred into WT neonates showed a significantly decreased proliferation upon antigen exposure in the liver, whereas there was comparable OTII proliferation in liver-draining lymph nodes and other lymphoid tissues in neonates and adults (Figs. S4a-b). Decreased proliferation persisted when antigen was presented endogenously by transgenically modified DCs (cDOG), suggesting an intrinsic immunosuppressive milieu in the neonatal liver rather than insufficient antigen availability and uptake (Fig. S4c). To directly test whether neonatal Tregs exert a suppressive effect on microbiota-induced Tconv activation, we made use of the DEREG mouse model, in which Tregs can be transiently depleted through the administration of diphtheria toxin (DT). Treg depletion during the first week of life strongly amplified hepatic CD4^+^ Tconv activation and frequency (Fig. 1l). Activated T cells upregulated CD69 and CXCR6 together with PD-1, resulting in an aberrant pro-inflammatory phenotype in the absence of Treg-dependent control, reminiscent of auto-aggressive liver T cells (Fig. 1l) (27). Collectively, these data demonstrate that the neonatal hepatic Treg wave is developmentally programmed, microbiota-independent, and functions to restrain the microbiota-dependent Tconv activation in the liver.

### The neonatal T cell wave is characterized by clonal proliferation and a shift to an effector phenotype of Treg and Tconv

We next investigated the antigen-dependence and clonal architecture of the early life hepatic T cell wave. Notably, without exposure to cognate antigen, there was no detectable neonatal burst of either Tregs or CD44^hi^ Tconvs in the liver of Ova-specific OTII Rag^−/−^ mice (Extended data 4d). These data led us to hypothesize that both T cell subsets primarily respond to cognate antigen presentation and to examine the clonal repertoire of the hepatic neonatal T cells embedded in the scRNA-seq dataset introduced in Fig. 1 (Fig. S3).

**Figure 2:**
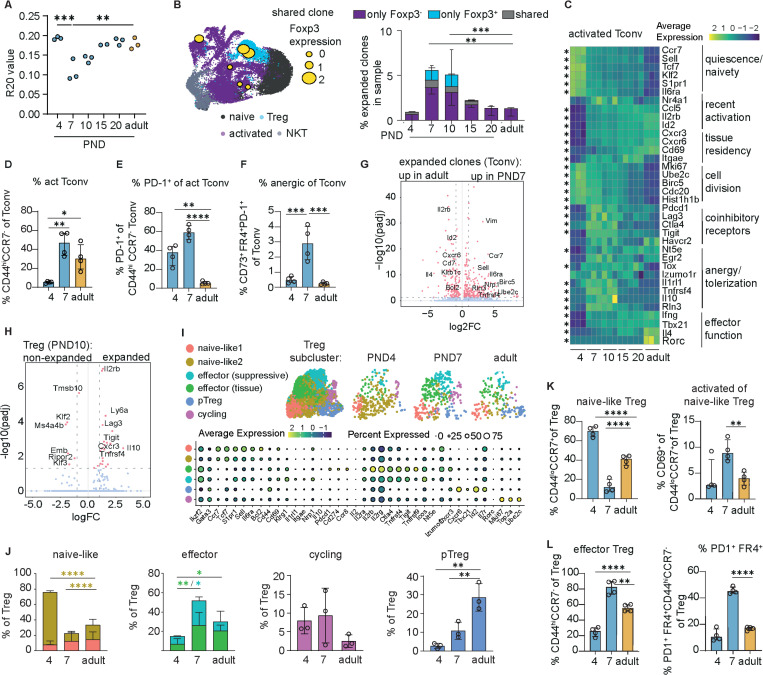
Clonally expanding Tregs and Tconv co-evolve into effector and anergy programs during in neonatal hepatic tissues (A) R20 score (defined by the fraction of unique clones, in descending order of frequency, which account for 20% of the sequenced repertoire) of clonal repertoire per mouse from VDJ scRNASeq. 2–3 mice per timepoint. One Way ANOVA + Dunnett’s multiple comparison to adult group. (B) % clones per mouse that are expanded (>1 cell of the same clonotype). 2–3 mice per timepoint composition of the expanded clones (either only Foxp3- cells in a clones purple,) only *Foxp3*^+^ (blue) and shared clones (both *Foxp3*^+^ and *Foxp3*^−^ cells in one clone). Two Way ANOVA + Dunnett’s multiple comparison to adult group. Representative depiction of a shared clone on a UMAP with cells depicted as yellow circles according to their *Foxp3* expression level). (C) Heatmap of canonical genes expressed over time in activated Tconv (purple cluster in A). Asterisks indicate DE gene expression between an early neonatal time point (PND4 or PND7) and adult time point. (D) % activated (CD44^hi^ CCR7^lo^) Tconv measured via flow cytometry, (E) % PD-1^+^ and % anergic (CD44^hi^ PD-1^+^ FR4^hi^ CD73^hi^) cells of activated Tconv, (F) % anergic of total Tconvs measured via flow cytometry. 3–4 mice per time-point from 1 representative experiment; One Way ANOVA + Tukey’s post hoc test. (G) Volcano plot with DE genes between Tconvs from expanded clones (>1 cell of the same clonotype) in 7-day old and adult mice. (H) Volcano plot with DE genes between expanded Foxp3+ cells (>= 2 cells per clone) and non-expanded Foxp3+ cells (<2 cells per clone) at PND10. (I) Reclustering of Tregs (blue cluster in A), bubbleplot displaying cluster marker genes used for identification, and UMAPs depicting composition at different time points after birth. (J) % of cells in a certain cluster within Tregs at PND4, PND7 and adult. One-Way ANOVA + Tukey’s post hoc test. (K) % CD44^lo^ CCR7hi of Tregs and CD69^+^ of CD44^lo^ CCR7^hi^ Tregs measured by flow cytometry and (L) % CD44^hi^ CCR7^−^ of Treg and PD1^+^ FR4^+^ of Treg measured by flow cytometry. One Way ANOVA + Tukey’s post hoc test. All experiments containing flow cytometry were done with the compared groups within 1 experiment processed on the same day. Asterisks indicate significance levels: * < 0.05, ** < 0.01, < 0.001 ***< 0.0001. All tests were performed in a two-sided manner.

In adult and very young (PND4) animals, the hepatic T cell population was almost exclusively polyclonal (Fig. 2a). In contrast, livers of neonatal mice after PND4 contained a markedly increased proportion of expanded T cell clones, coinciding with the neonatal T cell burst. These findings suggest that the expansion is at least partly clonal and driven by antigen-specific recognition. Interestingly, while there was minimal clonotype sharing between the Treg and the Tconv population in the liver of adult mice, mice at PND7 and older exhibited expanded shared clones, coinciding with the peak of the neonatal T cell expansion and consistent with the possibility that some of the hepatic neonatal Tregs are peripherally induced (Fig. 2b).

Consistent with the clonal repertoire data, activated Tconvs at PND4 displayed expression of naïve markers as well as cell cycle genes, indicating recent activation. By PND7, activated neonatal Tconvs acquired adult-like activation markers but also expressed inhibitory or anergy-associated genes (e.g., *Havcr2*, *Lag3*, *Tigit*, *Tnfrsf18* with reduced *Il2*), consistent with a controlled activation-to-anergy transition (Fig. 2c). Consistently, flow cytometric analysis of surface marker expression revealed an initial activation of Tconvs between PND4 and PND7 (Fig. 2d). Furthermore, a temporary increase of the fraction of PD-1^+^ cells and cells with an anergy-phenotype was observed within activated Tconv at PND7 (Fig. 2e-f). Comparing the phenotype of Tconv clones between neonate and adult revealed upregulated proliferation genes (*Mki67, Birc5, Ube2c*) alongside tolerance-linked regulators (*Tnfrsf4, Rln3, Nrp1*) in neonates, while in adults, the few expanded clones preferentially expressed tissue-resident and effector-associated genes (*Cxcr6, Klrb1c, Il4*; Fig. 2g).

Among neonatal Tregs, expanded clones preferentially expressed *Il10*, *Lag3*, *Tigit*, *Tnfrsf4* (OX40), highlighting a suppressive effector program of clonally engaged Tregs (Fig. 2h) and reflecting the strong transcriptomic changes in neonatal Tregs in the early postnatal period (Figs. 2i-j). Most hepatic Tregs bore a resting or “naïve-like” signature characterised by expression of *Tcf7, Sell*, *Ccr7* and others at PND4 (Figs. 2i-j), corresponding to a CD44^lo^ CCR7^+^ surface phenotype that shifted to a more activated phenotype at PND7 (Fig. 2k). The Treg expansion at PND7 was accompanied by a loss of the naïve-like Treg subset and a concomitant transition to an effector-Treg state (CD44^hi^ CCR7-; Fig. 2l) characterized by increased expression of *Pdcd1* (encoding PD-1), *Ctla4* and *Klrg1* (Fig. 2i-j) as well as surface protein expression of FR4 and PD1 (Fig. 2l). At both early time points (PND4, PND7) Tregs also showed increased expression of proliferation markers (*Mki67*, *Top2a*) suggesting that the increasing number of hepatic Tregs is at least partially due to local proliferation (Fig. 2j).

In addition, starting from PND7, a gradual accumulation of a Treg population marked by lower *Ikzf2* and higher *Cxcr6*, *Cxcr3*, *Tbx21* expression could be observed - these Tregs likely are a peripherally derived, tissue-imprinted Treg subset and represent a major population of adult hepatic Tregs, which was also evidenced by a higher proportion of CXCR6-expressing Tregs in adult livers (Fig. 2h-i; Fig. S4e). Finally, only a minor population of Tregs in the liver expressed *Rorc* (encoding RORγt) at any of the time points analysed, suggesting minimal RORγt^+^ pTreg contribution. (Fig. 2i; Fig. S4e).

Together, these data define the neonatal hepatic T cell wave as clonal and antigen-specific comprising both Tregs and Tconvs. Influx of early thymic Tregs led to differentiation of effector-suppressive tissue Tregs that establish a regulatory milieu in the neonatal liver. In parallel, naïve CD4^+^ T cells entering the liver undergo local activation. Interestingly, the clonal overlap suggests some naïve T cell conversion into peripherally induced Tregs, likely with distinct antigen specificities from the main Treg wave. The coexistence of thymic and peripherally induced tissue Tregs may therefore provide layered control over neonatal Tconv activation within the hepatic niche. By adulthood, this layered architecture condensed into a smaller CXCR6^+^ Treg compartment with reduced proliferative activity but maintained tissue residency, suggesting that the broad effector–suppressive spectrum of PND7 Tregs narrows into a stable, specialized pool reflecting the altered immune-functionality of adult hepatic tissue.

### Neonatal hepatic Treg expansion is driven by intrahepatic DC1-mediated antigen presentation

**Figure 3:**
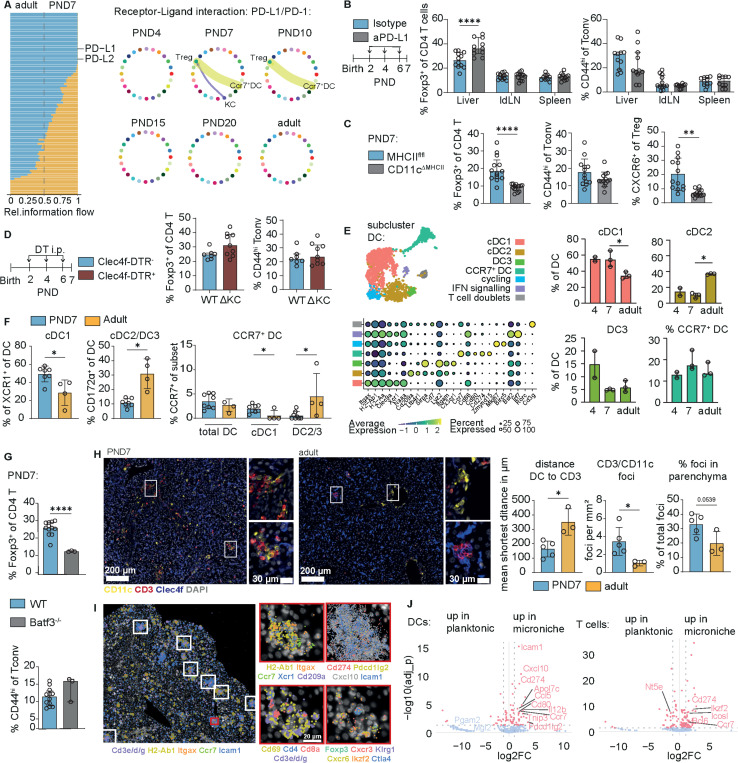
CCR7^+^ cDC1 assemble MHCII-dependent DC–T cell microclusters that enforce PD-L1/PD-L2-tuned tolerance (A) Overview of differentially expressed ligand receptor pairs predicted by CellChatDB between PND7 and adult CD45^+^ cells and circle plots displaying kinetic overview of predicted PDL1-PD1 interaction of the Treg cluster with other cell populations. Circles represent clusters/subsets from UMAP in Ext. Data 3c. (B) Experimental design for αPD-L1 blockade in neonatal WT mice and flow cytometry data of % of Treg within CD4 T cells as well as % of CD44hi within Tconv in liver, IdLN and spleen. Multiple Mann Whitney U tests. (C) Flow cytometry data of % of Treg within CD4 T cells as well as % of CD44hi within Tconv in liver in CD11cCre/+MHCIIflfl mice (CD11cΔMHCII) and their CD11c^+/+^MHCII^flfl^ (MHCII^flfl^)littermates. Mann Whitney U tests. (D) Experimental design for Kupffer cell (KC) depletion in neonatal Clec4fDTR mice and their WT litter mates; flow cytometry data of % of Treg within CD4 T cells. as well as % of CD44^hi^ within Tconv in liver. Mann Whitney U tests. (E) scRNASeq reclustering of dendritic cells from PND4 to adult hood with canonical marker genes displayed in bubbleplot and kinetics of the subclusters. One Way ANOVA + Tukey’s comparison. (F) Flow cytometry comparison of the liver DC compartment of neonatal WT and adult mice. Mann Whitney U tests. (G) Flow cytometry data of % of Treg within CD4 T cells as well as % of CD44hi within Tconv in liver of neonatal WT and BATF3^−/−^ mice at PND7, liver Treg frequency and CD44^hi^ Tconv frequency. Welch t-tests. (H) Multiplex immunofluorescence images and quantitative analysis of whole tissue slides; mean shortest distance between T cell (CD3+) and closest DC (CD11c^+^Clec4f^−^lba1^−^), density of manually counted CD3/CD11c foci in tissue and % of foci identified as in parenchyma by absence of vessels in close proximity. Welch t-tests. (I) Identification and characterization of microclusters in Xenium targeted spatial transcriptomics of neonatal liver (PND7). Left pictures shows indicated gene expression + T cell-DC clusters found by DBScan analysis highlighted in rectangles. Close-ups show the red highlighted cluster and expression of several transcripts of interest. (J) DE gene analysis of DCs and T cells inside and outside of microniches from Xenium panel. Top DE genes of cell type specific probes highlighted with labels. All experiments containing flow cytometry were done with the compared groups processed on the same day within 1 experiment (except BATF3−/− and WT pups). Asterisks indicate significance levels: * < 0.05, **< 0.01, *** < 0.001, **** < 0.0001. All tests were performed in a two-sided manner.

To directly address the mechanisms of induction and action of neonatal hepatic Tregs, we performed an unbiased ligand–receptor screen on the neonatal scRNA-seq kinetic (Fig. S5a-b), which highlighted cell-cell interactions guided by checkpoint axes. Specifically, the PD-L1 and PD-L2 signalling pathways were enriched in neonates, with expression of *Cd274* (PD-L1) on neonatal CCR7^+^ DCs and KCs, *Pdcd1lg2* (PD-L2) on neonatal NKs, ILCs and CCR7^+^ DCs, and strong *Pdcd1* (PD-1) expression on neonatal Tregs (Fig. 3a; Figs. S5c-d). In line with this, PD-L1 inhibition in neonates induced a shift in the regulatory tone: The frequency/number of Tregs increased, whereas Tconv numbers remained unchanged, leading to an increased Treg/Tconv ratio. (Fig. 3b; Figs. S5e).

Since our results demonstrated that neonatal Treg expansion was antigen-dependent (Fig. S5d), we hypothesised that either hepatic CCR7^+^ DCs or KCs act as principal APCs. To analyse their relative contribution, we made use of CD11c-Cre×H2-Ab1^fl/fl^ mice, which lack MHCII expression on DCs as well as Clec4f-DTR mice, in which KCs can be depleted. At PND7, a DC-specific MHCII deficiency resulted in a significant reduction in the Treg frequency as well as reduced Treg CXCR6 expression. Notably, the number of activated Tconvs was unchanged (Fig. 3c; Fig. S6a). In contrast, depletion of Kupffer cells or the deletion of MHCII on hepatocytes did not affect Treg frequencies nor Tconv activation status (Fig. 3d; Figs. S6b-c). Collectively, these results identify antigen presentation by DCs as the principal mechanism of neonatal hepatic Treg induction.

Transcriptome and flow-cytometry analyses identified a predominance of the cDC1 subpopulation in the neonatal liver, whereas the proportion of cDC2/DC3s increased after the postnatal period (Fig. 3e-f; Figs. S6d-g). Notably, despite its role in tolerance induction in the neonatal intestine(28, 29), we could not detect the presence of the tolerogenic RORγt^+^ DC subset in the neonatal liver (Fig. 3e). While the overall fraction of CCR7^+^ DCs remained unchanged between adults and neonates, the majority of the CCR7^+^ DC pool shifted from cDC1s in neonates to cDC2/DC3s in adults (Fig. 3f) suggesting that cDC1s may act as the principal APCs regulating the hepatic neonatal Treg wave. The decreased frequency of hepatic Tregs in cDC1-deficient BATF3 KO pups corroborated the role of cDC1s in regulating Tregs in the neonatal liver tissue (Fig. 3g). Crucially, postnatal liver T cell accumulation did not depend on migration of DCs to the LNs, as T cell density was not reduced in neonatal livers of CCR7^−/−^ mice (Fig. S7a).

In line with this, multiplex immunofluorescence microscopy revealed that T cells in neonatal liver tissue were organised in microclusters and colocalised with CD11c^+^ Iba1^−^ Clec4f^−^ DCs as well as Tconvs (Fig. 3h). These DC-T cell microclusters were significantly more prevalent in neonatal compared to adult livers. Moreover, they had a distinct localisation: in adult tissues the DC-T microclusters were mainly confined to periportal areas, while they were abundant in the liver parenchyma of neonates (Fig. 3h; Fig. S7). Crucially, spatial transcriptomics confirmed co-localization of DCs, Treg and Tconv in microclusters in the neonatal liver (Fig. 3i; Figs. S7 a-e). DCs in neonatal liver microniches expressed significantly more *Ccr7, CD80, Pdcd1-lg2, Cd274* corresponding to CCR7^+^ DCs (Fig. 3j; Fig. S7f), while T cells showed a significant enrichment of Helios (*Ikzf2* transcript) within the clusters (Fig. 3j; Figs. S7g-i). Notably, DC-T cell clusters in neonatal livers showed a high level of *Cxcr3* transcripts and its ligand *Cxcl10* suggesting a chemokine-dependent origin of these cell clusters (Fig. 3i; Figs. S7 d-e).

Together, the presented results identify anatomically discrete, cDC1-anchored regulatory clusters in the neonatal liver that (i) depend on MHCII on CD11c^+^ DC, (ii) assemble CCR7^+^ cDC1–Treg–Tconv triads in parenchyma and near portal tracts, and (iii) use PD-L1/PD-L2 checkpoints to stabilize tolerance as the liver transitions postnatally. These data align with postnatal portal-area tissue remodelling and the zone-specific maturation of hepatic stromal compartments, providing a structural basis for immune organisation in early life (30, 31).

### The neonatal liver environment confers tolerance to *in situ* activated T cells with consequences for infection

**Figure 4:**
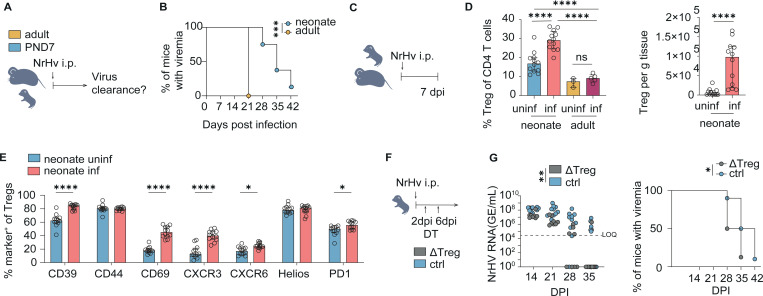
Tolerance-biased neonatal liver delays viral clearance in a hepatitis C model despite robust T-cell activation. (A) Experimental design of NrHV infection model. (B) Clearance rates of neonatal mice infected at PND7 and infected adult mice. 5–10 mice from 1 adult and 2 independent neonatal experiments. Kaplan Meier survival curve + log-rank test. (C) Experimental design of NrHV infection model for acute infection flow cytometry analysis. (D) % Treg of CD4 T cells in liver tissue of uninfected and infected neonates and adults 7 days post infection (dpi) and Treg density in liver tissue of infected and uninfected neonatal mice. Flow cytometry data of 11–12 neonatal mice from two independent experiments and 3–5 adult mice from 1 experiment. One Way ANOVA + Holm-Sidak multiple comparison tests. Treg density: Mann Whitney U test. (E) Phenotypic markers of liver Treg in neonatal uninfected and infected and adult infected mice 7 days post-infection measured by flow cytometry. 12 neonatal mice per group from 2 independent experiments. Multiple Mann Whitney U tests + Holm-Sidak multiple comparison correction. (F) Experimental design of Treg depletion during NrHV infection in neonates. (G) Viremia levels in NrHV infected Foxp3-DTR mice receiving 2 doses of diphtheria toxin (DT) and infected WT control neonates 2 and 6 dpi. 8–10 neonatal mice per group pooled from 1–2 independent experiments. Two Way ANOVA (blood viremia levels) and Kaplan Meier survival curve + log-rank test. Asterisks indicate significance levels: * < 0.05, ** < 0.01, *** < 0.001 **** < 0.0001. All tests were performed in a two-sided manner.

We next asked whether this strong immunoregulatory environment in the neonatal liver tissue may be detrimental during hepatotropic infection. Using the Norway rat hepacivirus (NrHV) model (a mouse HCV surrogate (32)), we infected PND7 neonates and adults. Despite the higher baseline density of Tconv and CD8^+^ T cells in neonatal liver tissues (see Fig. 1), viral clearance was delayed in neonates relative to adults (Figs. 4a-b; Fig. S8a). Seven days post-infection, neonates displayed a robust, liver-specific induction of Tregs, which was not seen in adult mice. Neonatal Tregs also showed increased expression of effector and suppressive markers (Figs. 4c-d; Fig. S8b). Nevertheless, neonatal Tconv and CD8^+^ T cell densities in the neonatal livers increased strongly during the course of infection, and upregulated activation markers (Figs. S8c-d). However, Tconvs showed decreased effector marker expression in infected neonatal mice when compared to adult infected mice (Fig. S8c), while CD8^+^ T cells were activated to the same level in neonatal and adult infected mice (Fig. S8d). To test whether neonatal Tregs causally influenced the antiviral host response kinetic, we transiently depleted Tregs in neonates shortly after infection (Figs. 4f-g) using a previously established protocol to induce virus clearance in chronically infected adult mice (33). Treg depletion led to a decreased viral load and accelerated viral clearance, consistent with a functional role of the developmental Treg wave in modulating early antiviral immunity (Figs. 4f-g). Taken together, the neonatal hepatic niche enforces tolerance on freshly activated T cells, beneficial under commensal colonization to prevent immunopathology (17, 18), yet detrimental during hepatotropic virus infection, where it tempers effector function and delays viral clearance.

### Early-life Treg programs shape adult susceptibility to diet-induced Metabolic Dysfunction-Associated Steatotic Liver Disease (MASLD)

**Figure 5:**
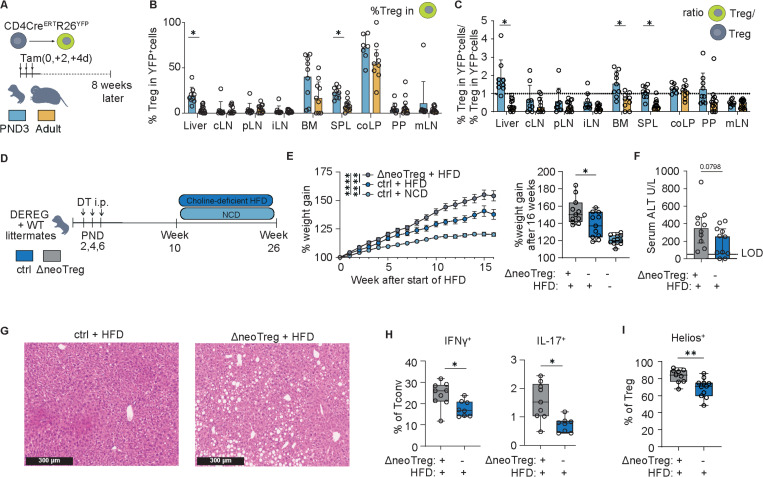
Early-life Treg programs shape adult susceptibility to diet-induced MASLD (A) Experimental design for time-stamping experiment of T cells in CD4^CreERT^ R26^YFP^-model. (B) % of Treg in YFP^+^ CD4 T cells 8 weeks after timestamping per organ and (C) ratio of Treg in YFP^+^ and YFP^−^ CD4 T cells. Mann Whitney U tests. (D) Experimental design of diet induced MASLD experiment with neonatal Treg depletion in DEREG mice and WT littermates. 8–11 mice from each group from 5 independent experiments. Only litters containing both male DEREG and WT littermates were used for HFD analysis. (E) % weight gain during the HFD treatment in neonatally transiently Treg depleted male mice, their littermates and normal chow diet (NCD) fed control mice from (d) (parametric, paired mixed-effects analysis + Holm Sidak multiple test correction) and % weight gain at the end of the experiment (Unpaired t-test). (F) Serum ALT levels in male mice from (d). Unpaired t-test. (G) representative H&E stainings of liver tissue from (H) IFNy^+^ and IL-17^+^ of Tconv after restimulation with PMA/lonomycin and (I) % of Heliost^+^ cell within Tregs measured by flow cytometry. All experiments containing flow cytometry were done with the compared groups processed on the same day within 1 experiment. Asterisks indicate significance levels: * < 0.05, <0.01, ***<0.001 **** < 0.0001. All tests were performed in a two-sided manner.

To test whether early-life Tregs seed long-lived regulatory compartments, we lineage-tagged CD4^+^ T cells either in neonates or adults using CD4-CreERT2;Rosa-YFP mice and analyzed them 8 weeks later (Fig. 5a). While there was no difference in the overall proportion of YFP^+^ CD4^+^ T cells in the liver (Fig. S9a), neonatal timestamping yielded a disproportionately high fraction of YFP^+^ Tregs in adult liver, whereas adult timestamping contributed minimally to the hepatic Treg pool (Fig. 5b). Comparing YFP^+^ vs. YFP^−^ compartments showed that early-life-tagged CD4^+^ cells were strongly biased toward a Treg fate in the liver, consistent with (i) long-term retention of neonatal thymic Tregs and/or (ii) preferential pTreg differentiation from tissue-imprinted effectors within the neonatal hepatic niche (Fig. 5c). We next asked whether these neonatally generated persisting Tregs had lasting consequences. For this, we timestamped neonatal CD4^+^ T cells and challenged the mice as adults with a choline-deficient, high-fat diet (HFD) that drives steatosis and metabolism-associated steatohepatitis (MASH) over 16 weeks (Fig. S9b). In this model, subsets of effector Tregs contribute to fibrogenic programs under metabolic stress (34). Across groups, high-fat feeding increased the overall Treg share in liver CD4^+^ T cells and selectively expanded Helios^+^ Tregs approximately 3-fold. Lineage tracing showed that this expansion also occurred within neonatally derived (YFP^+^) cells, indicating that early-life–imprinted Tregs remain responsive in adulthood (Fig. S9b). Finally, to definitively determine whether the neonatal Treg wave had functional relevance for the development of metabolic liver disease in later life, DEREG neonates were transiently depleted of Tregs during the first postnatal week and subjected to HFD for 16 weeks as adults (Fig. 5d). Neonatally depleted males showed a modest but significant excess weight gain, trending alanine aminotransferase (ALT) levels, and increased histological steatosis, while glucose tolerance was unchanged (Fig. 5e-g; Fig. S9c). Female mice displayed a MASLD phenotype that was independent of neonatal Treg depletion. (Sig. S9d). The pathological MASLD phenotype in neonatally depleted males was accompanied by increased levels of Th1 and Th17 effector cells, indicating a pro-inflammatory skew (Fig. 5h). Notably, the Helios^+^ Treg fraction increased in the HFD group, whereas the total Treg frequency within CD4^+^ T cells was unchanged between the DEREG and WT HFD groups, suggesting an altered Treg composition rather than an absolute loss after early-life Treg depletion (Fig. 5i). Taken together, neonatal Treg programs durably shape the adult hepatic T cell compartment and modulate the susceptibility to metabolic liver disease. Early-life generated T cells preferentially populate the adult liver Treg niche, transient neonatal Treg loss primes male-biased hepatic steato-inflammation under dietary stress, and dietary challenge amplifies a Helios^+^ Treg subset that includes neonatally derived cells.

## Discussion

Our data identify a disproportionately large CD4 T cell pool in the neonatal liver, compared with other organs, characterized by a pronounced Treg wave that is independent of the microbiota, and a concurrent accumulation of activated Tconv modulated by microbial exposure. TCR/clonotype analyses and transcriptomic/phenotypic characterization of Tregs in early life hepatic tissue point to a biphasic program: an early, developmentally hard-wired tTreg wave that arrives in a naïve-like state and differentiates *in situ* into tissue effector Tregs, followed by a secondary enrichment of peripherally induced Tregs consistent with an anergy-to-pTreg maturation trajectory(35, 36). Conceptually, the neonatal hepatic Treg wave fits the broader pattern of early-life regulatory responses at barrier sites. In lung and skin, microbiota-dependent Treg accumulations arise later in the pre-weaning period (around postnatal day 8 in lung and day 13 in skin, respectively) and have been suggested to restrain local inflammatory priming(14, 15). In contrast, the neonatal liver hosts an earlier, microbiota-independent expansion, suggesting a developmentally hard-wired regulatory program that precedes and complements microbiota-dependent regulation at barrier tissues. In our data, neonatal Tconv responsiveness tracks the level of microbial exposure, whereas the Treg peak is exposure-insensitive; we interpret this as a division of labor, in which endogenous/self-antigen preferentially sustains Tregs while microbe-associated signals “titrate” Tconv activation. A developmentally programmed, microbiota-independent Treg wave may be essential to calibrate the transition to highly variable postnatal exposure to microbial and dietary antigens.

Functionally, this program maximizes regulatory bandwidth at a time when the peripheral CD4^+^ pool is dominated by recent thymic emigrants (RTEs), a population more self-reactive and less stringently tuned than adult naïve T cells(1–4). Within this context, the successive Treg waves likely differ in TCR specificity and function: early tissue Tregs provide rapid local control of Tconv activation as polyreactive RTE-Tregs and non-Tregs first enter the periphery, whereas later Tregs, of either thymic or peripheral origin, consolidate longer-term protection from immune-mediated pathology, including metabolic liver disease.

Where first antigen encounters occur is pivotal: During suckling stage, luminal antigen access to the immune system of the gut is restricted(10, 13), yet the portal circulation can still ferry self-derived metabolites and microbiota-derived cues to the liver, in addition to abundant self-derived signals generated by the metabolically active parenchyma. At present, we cannot distinguish whether Tconv tuning is driven primarily by cognate microbial antigens or by non-antigenic PAMPs. A parsimonious explanation is that immature enterocytes engage in bulk endocytosis/macropinocytosis, allowing microbial material, including both antigens and PAMPs, to reach the portal circulation despite reduced classical macromolecule uptake, a process often discussed under the umbrella of “gut closure”(37, 38). Within this framework, our data characterize a neonatal hepatic environment in which antigen presentation by cDC1s results in a tolerance-promoting microenvironment along the gut–liver axis, consistent with CD4^+^ T cell hyporesponsiveness and the inhibition of self-reactivity(36, 39).

At the cellular wiring level, our imaging and perturbation experiments show that CCR7^+^ cDC1 nucleate APC–T cell clusters that act as tolerogenic foci in the neonatal liver. Within these sites, PD-L1/PD-L2 checkpoints constrain Tconv activation while permitting robust Treg accumulation. Anti-PD-L1 treatment therefore results in selective Treg accumulation without unleashing Tconvs, consistent with differential checkpoint sensitivity of the two compartments rather than a single dominant effector mechanism. This aligns with the broader view that Treg control emerges from precise positioning and multiple, context-dependent mechanisms rather than one canonical pathway(40). Notably, while RORγt^+^ DC populations are pivotal in intestinal tolerance, we do not detect a RORγt^+^ DC contribution in the neonatal liver; instead, cDC1 dominate the activated CCR7^+^ pool. This organ-specificity fits with current syntheses that place RORγt^+^ DCs at the center of gut tolerance but not in every tolerogenic niche(41).

The anatomical microenvironment of these interactions is unique for hepatic tissue in early life. We observe cDC1-anchored T cell microniches not only in periportal areas but also widely distributed across the parenchyma, an anatomical arrangement that is lost in adulthood. Work on neonatal liver development supports a window in which immune and stromal programs are broadly distributed across the parenchyma and remodel toward the adult architecture around weaning, providing a structural rationale for elevated CD4^+^ density and Treg skew in neonates(31, 42, 43). Within this setting, spatial transcriptomics reveals that neonatal DC-T cell clusters are enriched for transcripts associated with leukocyte migration, including *Ccr7*, *Cxcr3* and *Cxcl10*, and colocalize with *Icam1*^+^*Cxcl10*^+^ structural cells that are distinct from hepatocytes. These features support a chemokine- and adhesion molecule-dependent origin of the microniches and resemble Th1 response-enhancing CXCL10^+^ activation niches described in other tissues(44, 45). At the same time, their dispersed, T cell–dominated architecture and lack of B cell organization distinguish them from the B cell-rich tertiary lymphoid structures that can arise in chronically inflamed livers and other organs(21, 23, 24).

Within this framework, the balanced activation we observe, characterised by clear CD4^+^/CD8^+^ responses to microbial exposure but slower pathogen control under hepatotropic virus challenge, fits a tolerance-biased set point in neonates that prioritizes tissue protection while accepting the trade-off of delayed pathogen clearance(46). Transient disruption of neonatal regulation imprints long-lasting vulnerability patterns in adulthood, highlighting its non-redundant functionality and suggesting that the composition and education of the neonatal T cell pool, more than sheer numbers, forecast later liver inflammatory risk(47). Paediatric livers exhibit strong tolerogenic features, and early-life hepatotropic virus infections more readily become chronic. Our data provide a mechanistic basis for neonatal hepatic tolerance: a CCR7^+^ cDC1-anchored, regulatory immune architecture that skews activation outcomes and Treg differentiation along the gut–liver axis(36, 47). In humans, this may offer a plausible explanation for the age-dependent pathology of hepatitis B virus: newborns typically develop chronic infection, whereas adults more often mount acute, self-limiting disease, consistent with a neonatal liver environment that dampens Tconv/CD8 priming and cytotoxic function, favoring viral persistence.

Notably, in chronic metabolic disease the Treg program can become maladaptive. In mouse MASH, hepatic Tregs expand and adopt an activated CCR8^+^/ST2^+^/KLRG1^+^ state that promotes amphiregulin-dependent stellate-cell activation and fibrosis(34). Although the CCR8^+^/ST2^+^/KLRG1^+^ Tregs reported in MASLD/MASH display hallmarks of tissue Tregs, that overlap with features we also observe in neonates, our findings indicate that neonatally derived tissue Tregs are protective in the long term. Tissue Treg pools in non-lymphoid organs, including in the liver, are now understood to be shaped by transient multi-tissue migration and a conserved residency program rather than life-long persistence of a fixed neonatal cohort(47).

Two non-exclusive factors may reconcile this with reports of pathogenic CCR8^+^/ST2^+^/KLRG1^+^ Tregs in established disease. First, kinetics: neonatal tissue Tregs are in place at baseline, shaping responses from the onset, whereas MASLD-associated Tregs are recruited or reprogrammed during disease and may act at later, injury-rich timepoints, where their repair/EGF-family programs can inadvertently foster fibrosis. Second, TCR specificity: neonatal tissue Tregs are likely selected to distinct antigenic landscapes (self, perinatal antigens) that differ from the damage- and lipotoxicity-derived antigens encountered in MASLD. Thus, despite similar surface programs, their TCR repertoires and instructive cues may drive divergent functions. This framework predicts that early-life Treg ontogeny/timing and TCR repertoire, rather than phenotype alone, will stratify MASLD risk and outcomes.

## Supplementary Material

Supplement 1

## Data Availability

scRNASeq raw FASTQ data has been deposited in ENA with accession code PRJEB104713. The processed data is available in Zenodo (DOI:10.5281/zenodo.17940609, available from 31/12/2025). 10X Genomics Xenium raw and processed data have been deposited in Zenodo (DOI:10.5281/zenodo.17940609).
