# Supplementary material for: Neonatal liver niches program T cell tolerance
Source: bioRxiv. 2026 Jan 13:2026.01.13.698576. Preprint. [Version 1] doi: 10.64898/2026.01.13.698576 (PMC12871242; doi:10.64898/2026.01.13.698576)
Supplement: Supplement 1 [file NIHPP2026.01.13.698576v1-supplement-1.pdf]

bioRxiv preprint doi: https://doi.org/10.64898/2026.01.13.698576; this version posted January 13, 2026. The copyright holder for this preprint (which was not certified by peer review) is the author/funder, who has granted bioRxiv a license to display the preprint in perpetuity. It is made available under aCC-BY 4.0 International license.

Supplementary files

Materials and Methods

Supplementary Figure 1-9

bioRxiv preprint doi: https://doi.org/10.64898/2026.01.13.698576; this version posted January 13, 2026. The copyright holder for this preprint (which was not certified by peer review) is the author/funder, who has granted bioRxiv a license to display the preprint in perpetuity. It is made available under aCC-BY 4.0 International license.

Methods:

All animal experiments were performed in compliance with the German animal protection law (TierSchG) and approved by the local animal welfare committees, the Landesamt für Verbraucherschutz und Ernährung (LAVE), North Rhine Westfalia (M2025-728, 81-02.04.2017, A460; 81-02.04.2018.A386), Lower Franconia Government and Regierungspräsidium Freiburg, Freiburg (X-20/05F), the Cantonal Veterinary Commission of the Canton of Bern, Switzerland (BE-132/23). Viral infection experiments were performed under specific pathogen-free conditions at the Institute for Animal Studies of Albert Einstein College of Medicine using C57BL/6 or FOXP3-DTR mice (Jackson Laboratory). All mouse experiments were conducted in accordance with the NIH Guide for the Care and Use of Laboratory Animals and approved by the Albert Einstein College of Medicine Institutional Animal Care and Use Committee (protocol number 00001108).

C57BL/6J wild-type (WT), B6.Cg-Tg(TcraTcrb)425Cbn Rag1$^{tm1Mom/J}$ (OTII Rag-/-), C57BL/6-Tg(Foxp3-HBEGF/EGFP)23.2Spar/Mmjax (DEREG), B6-Clec4^{ftm1Ciphe} (Clec4f-DTR) mice, B6.129S(C)-*Batf3*^{tm1Kmm}/J, Tg(Itgax-cre)1-1Reiz x H2-Ab1$^{b\text{-}tm1Koni}$, Tg(Cd4-cre/ERT2)11Gnri x B6.129X1-*Gt(ROSA)26Sor*$^{tm1(EYFP)Cos}$/J , Tg(Alb1-cre)7Gsc x H2-Ab1$^{b\text{-}tm1Koni}$, CCR7$^{gfp/gfp}$ (CCR7 KO) were bred locally and held under specific pathogen–free (SPF) or germ-free (GF) conditions at the Institute of Laboratory Animal Science at RWTH Aachen University Hospital, or Institute for Hygiene and Microbiology Würzburg. C57BL/6NTac wildlings were bred locally at the animal facility of the Medical Center – University of Freiburg, Germany. C57BL/6NTac murine pathogen free (MPF) control mice were originally purchased from Taconic Biosciences, subsequently bred locally and housed under SPF conditions at the animal facility of the Medical Center - University of Freiburg, Germany. Wildlings and C57BL/6NTac MPF mice were age matched for all experiments.

In vivo models

Perinatal Treatment with Broad-spectrum Antibiotics: Pregnant dams were treated from E14.5 to PND7 of the offspring with ampicillin (1 mg/mL), neomycin (0.5 mg/mL), vancomycin (1 mg/mL) ad libitum in the drinking water. Additionally, dams were gavaged i.g. once per day with 0.2 mg/g body weight metronidazole dissolved in drinking water for depletion of anaerobic bacteria as metronidazole could not be delivered ad libitum due to the bitter taste of this antibiotic(*48*). The antibiotic-containing drinking water was prepared and exchanged daily.

Reversible Colonisation with E. coli HA107: Germ-free mice were either colonised during pregnancy (gestational colonisation of the dam; GC) or in the first week after birth at PND3 and PND5 (Postnatal colonisation of the pups; PC) the E. coli strain HA107 that is auxotrophic for the amino acids meso-diaminopimelic acid and D-alanine(*49*). HA107 was grown in 200 mL LB medium containing 100 µg/mL meso-diaminopimelic acid and 400 µg/mL D-alanine overnight at 37 °C on a shaking incubator and washed twice in sterile PBS. For gestational reversible colonisation, pregnant germ-free dams were gavaged i.g in gnotobiotic isolators as previously described(*50*) at E7, E9, E11 and E13 of the pregnancy with 10^{10} CFU. For

bioRxiv preprint doi: https://doi.org/10.64898/2026.01.13.698576; this version posted January 13, 2026. The copyright holder for this preprint (which was not certified by peer review) is the author/funder, who has granted bioRxiv a license to display the preprint in perpetuity. It is made available under aCC-BY 4.0 International license.

postnatal reversible colonisation, germ-free pups were colonised with $7x10^8$ CFU HA107 at PND3 and 10^9 at PND5 by gavage i.g. within a sterile environment under the laminar flow hood.

E.coli outer membrane vesicles (OMV) Treatment: E. coli OMV were prepared by the lab of Nelson Gekara at Umea University, Sweden as described by(*51*). OMVs were administered i.g. (25 mg in 20 µL per pup) to 10-day old pups in PBS eight hours before analysis.

Treg depletion: Diphtheria toxin (Merck, No. 322336) was used to deplete regulatory T cells in neonatal DEREG mice. DEREG and WT mice were injected intraperitoneally with 25, 50 and 75 ng DT respectively on PND (Postnatal day) 2, PND4 and PND6 in 10-20 µL PBS.

Antibody Treatments: *α*PDL1 blockade antibody (Clone: 10F.2H11; InVivoMAb, Bioxcell) was applied to WT pups at PND2, PND4 and PND6 i.p. 10-20 µL in sterile PBS. Littermates were treated with isotype-matched control antibody (InVivoMAb rat IgG2b isotype control, *α*-keyhole limpet hemocyanin, Bioxcell).

KC depletion: Diphtheria toxin (Merck, No. 322336) was used to deplete regulatory T cells in neonatal B6-Clec4ftm1Ciphe (Clec4f-DTR) mice. DTR+ and DTR- mice were injected intraperitoneally with 25, 50 and 75 ng DT respectively on PND (Postnatal day) 2, PND4 and PND6 in 10-20 µL PBS.

NrHV infection: Norway rat hepacivirus (NrHV) stocks were generated from a cDNA clone as described previously(*32, 52*). Adult C57BL/6 mice (8–10 weeks) were infected intravenously via the retro-orbital sinus; neonatal mice were infected intraperitoneally at postnatal day (PND) 7 with 10^4 genome equivalents (GE) NrHV in sterile PBS. Serum was collected at the indicated time points and viral RNA copies were quantified by RT–qPCR targeting the NrHV NS3 region using an in-vitro-transcribed RNA standard curve, essentially as described(*32*).

For Treg depletion during infection, neonatal FOXP3-DTR and WT littermate controls received diphtheria toxin (25 ng/g body weight in 50 µL PBS, i.p.) at 2 and 6 days post infection. Mice were sacrificed at 7 days post-infection. Livers and spleens were collected into 10 mL and 7 mL of RPMI supplemented with 10% FCS, T cells were isolated immediately. Tissues were minced and digested in HBSS-based digestion buffer containing 0.01% collagenase IV, 40 mM HEPES, 2 mM $CaCl_2$, and 2 U/mL DNase I for 12–15 min at 37°C, followed by mechanical homogenization. Subsequent lymphocyte isolation was performed according by density gradient according to “liver cell isolation”. Viability staining was performed first using the Zombie NIR Fixable Viability Kit (BioLegend). Surface staining was conducted at 20°C for 15 min. Intracellular staining was performed following permeabilization using the FOXP3/Transcription Factor Staining Buffer Set (eBioscience), with a 20 min permeabilization at 4°C followed by a 30 min intracellular stain at 4°C. After staining, cells were fixed in 4% paraformaldehyde (PFA).

bioRxiv preprint doi: https://doi.org/10.64898/2026.01.13.698576; this version posted January 13, 2026. The copyright holder for this preprint (which was not certified by peer review) is the author/funder, who has granted bioRxiv a license to display the preprint in perpetuity. It is made available under aCC-BY 4.0 International license.

Time Stamping: 50 µg/g body weight tamoxifen (Sigma) was applied three times in 7.5 µg/µL in Clinoleic Solution (Baxter) either to 8-12 week old mice or to neonatal mice on PND3, PND5 and PND7 after birth by intragastric gavage.

Choline-Deficient High-Fat Diet: For induction of metabolic dysfunction associated with steatotic liver disease after the different neonatal interventions, mice were placed on a choline-deficient high-fat diet (HFD; (cat. no. D05010402; Research Diets) at the age of 10 weeks for a period of four months. On the last day of the dietary treatment, oral glucose tolerance was tested by fasting the mice for 6 h. Baseline glucose levels were measured with a standard blood glucose monitoring device (AccuCheck) from a drop of tail vein blood. Mice were then gavaged i.g. with glucose (2 mg/g body weight) dissolved in drinking water. Blood glucose levels were measured after 15 min, 30 min, 60 min and 120 min.

Isolation of liver immune cells: Livers were perfused with PBS/3 % FCS via the vena cava; the gall bladder was removed, and the liver was weighed and cut into pieces. Liver pieces were either digested in 10 mL of RPMI with Liberase*TM* (Roche; 30 µg enzyme/mL digestion solution) and DNase (Roche; 10 µg enzyme/mL digestion solution) for 30 min at 37 °C in a shaking water bath. Tissue remnants were homogenised by processing through a 10 mL syringe, filtered through a 100 µM cell strainer, and rinsed with 10 mL PBS/FCS to retain a single-cell suspension, which was centrifuged for 8 min at 400× g to pellet the harvested cells. In some experiments liver cells were isolated by mechanical dissociation (for scRNASeq and in case of CD62L flow cytometry staining): up to 400 mg of liver tissue was placed in the chamber of the Tissue Grinder device with 800 µL of PBS/3 % FCS (FFX Technologies), and mechanically dissociated using the Liver Program of the device twice; the tissue grinder tube was then centrifuged for 5 min at 400× g to pellet the harvested cells.
The obtained pellet from both methods was then resuspended in 4 mL of a 40 % solution of phosphate- buffered PBS/Percoll polymer in RPMI/FCS and carefully layered over 4 mL of a 70 % solution of phosphate-buffered Percoll polymer (Cytiva). Density gradient centrifugation was performed for 25 min at room temperature at 700× g without brake and acceleration set to level 3. After the gradient, the purified immune cells were collected from the interphase in a volume of 1.5 mL. After washing in 13 mL PBS/FCS and centrifugation at 800× g for 8 mins, cells were resuspended in an appropriate volume and transferred to the staining plate to perform flow cytometry staining.

Isolation of other organ immune cells: Spleens were brayed with the plunger of a syringe in 1 mL PBS/FCS and filtered through a 100 µm mesh into a 15 mL tube. 10 mL red blood cell lysis buffer was added. After 8 min of incubation, cells were centrifuged for 8 min at 400× g. The supernatant was discarded, and cells were washed once in 5 mL PBS/FCS and centrifuged for 5 min at 400× g before flow cytometry staining. **Celiac, portal and inguinal lymph nodes** were dissected from the tissue, and remaining fat was removed. Peyer's patches were removed from the small intestine by cutting them out with ine scissors and collected in PBS/FCS. Lymph nodes and Peyer's patches were then transferred and digested in 1 mL of RPMI containing 50 µg/mL Liberase*TM* (Roche) and 10 µg/mL DNase (Roche) in

bioRxiv preprint doi: https://doi.org/10.64898/2026.01.13.698576; this version posted January 13, 2026. The copyright holder for this preprint (which was not certified by peer review) is the author/funder, who has granted bioRxiv a license to display the preprint in perpetuity. It is made available under aCC-BY 4.0 International license.

10% FCS/RPMI for 40 min in a shaking water bath at 37 °C. After 20 min of incubation, the tissue was mechanically disrupted by pipetting up and down. At the end of the incubation, cells were filtered through a 100 µm filter (Falcon), washed, and then centrifuged for 5 min at 400× g before flow cytometry staining. **Blood** was collected in EDTA-coated tubes (Sarstedt) filled with 500 µL of 20 mM EDTA in PBS. For red blood cell lysis, the cell suspension was transferred into 10 mL RBC lysis buffer (150 mM NH_4Cl, 10 mM $KHCO_3$, 0.1 mM EDTA-Na_2 in distilled water [dH2O]), mixed and incubated for 8 min at room temperature (RT). Cells were immediately centrifuged for 8 min at 400× g, washed in 5 mL PBS/FCS, and centrifuged again for another five minutes at 400×*g* before staining. **Bone marrow** cells were isolated by dissection, cleaning and flushing it out from one tibia for adult mice or both tibiae for neonatal mice using 2 mL of PBS/3% FCS. The cell suspension was centrifuged for 5 min at 400×g, then resuspended in 2 mL red blood cell lysis buffer and incubated for 2 min at room temperature to lyse erythrocytes. Next, cells were centrifuged (5 min at 400×g, 4C), washed twice in PBS/FCS and stained. **Small intestinal lamina propria:** After removal of the small intestine from the sacrificed mouse the adhering fat was removed and the intestine was cut open longitudinally. Intestinal content was removed by shaking in 10 mL PBS in a petri dish. The intestinal tissue was then shaken at 37 °C and 225 rpm for 20 min in 2 mM EDTA in 20 mL Hank's balanced salt solution (Gibco)/3 % FCS, shaken vigorously by hand at the end of the 20 min incubation period and then filtered through a 100 µM cell strainer (Corning). The intestinal tissue remaining in the filter was transferred to a new tube with 2mM EDTA in 20 mL Hank's balanced salt solution (Gibco)/3%FCS and the incubation step was repeated. The remaining intestinal tissue was cut into small pieces and then digested for 40 min at 37 °C and 225 rpm in a shaking water bath in 10 mL RPMI/10%FCS containing LiberaseTM (Roche; 30 µg enzyme/ mL digestion solution) and DNase (Roche) (10 µg enzyme/ mL digestion solution). 20 min into the incubation period, tubes were additionally shaken by hand to improve tissue disintegration. After 40 min, the digestion was stopped by adding 30 mL of PBS/3%FCS and cells were centrifuged for 10 min at 800 x g and the supernatant was discarded. For enrichment of immune cells, a density gradient was performed similar to the liver immune cell isolation. **Colon lamina propria:** After removal of the colon from the sacrificed mouse, the adhering fat and faeces were removed, and the colon was cut open longitudinally and washed in a petri dish with 15 mL cold HBSS/3 % FCS. The colon tissue was then shaken at 37 °C and 225 rpm for 20 min in 2 mM EDTA in 20 mL HBSS/3 % FCS, shaken vigorously by hand at the end of the 20 min incubation period, and then filtered through a 100 µM cell strainer to ensure the removal of epithelial cell lining. This was repeated twice in total. The remaining colon tissue was cut into small pieces and then digested in Collagenase D (12.5 µg/ml, Roche), Dispase (10µg/ml, Gibco)) and DNase (10 µg/mL; Roche) in 10 mL RPMI/3 % FCS at 37 °C for 45 min. At the end of the incubation period, tubes were vigorously shaken by hand to improve tissue disintegration, filtered through a 100 µM cell strainer, and then directly centrifuged for 8 min at 400×g at 4 °C. For enrichment of immune cells, a density gradient was performed similar to liver immune cell isolation.

Flow cytometry staining

After obtaining a single cell suspension, cells were transferred into a 96 well plate for staining and incubated with TruStainFcx (1/100 diluted PBS/FCS; Biolegend) for 10 min at 4 °C to block unspecific binding by the macrophage receptors CD16 and CD32. For CCR7 detection on T cells, CCR7 antibody was added 1:200 and cells were incubated 1 h at 37 °C. Cells

bioRxiv preprint doi: https://doi.org/10.64898/2026.01.13.698576; this version posted January 13, 2026. The copyright holder for this preprint (which was not certified by peer review) is the author/funder, who has granted bioRxiv a license to display the preprint in perpetuity. It is made available under aCC-BY 4.0 International license.

were centrifuged at 400 x g, 4 °C for 5 min. Fixable viability dye 780 (ebioscience; 1/1000 diluted in PBS), Zombie UV or ZombieNIR fixable viability dye (Biolegend; 1/1000 diluted in PBS) stained for 30 min at 4 °C in a total volume of 100 µL per well, then cells were centrifuged at 400 x g, 4 °C for 5 min. Surface staining was done in 50 µL of antibody staining mix for 20-30 min at 4 °C. Cells were fixed with Foxp3 staining kit (ebioscience; for detection of transcription factors) or with Cytofix/Cytoperm kit (BD Biosciences; for the concomitant detection of cytosolic proteins such as cytokines or GFP with transcription factors) for 20 min at RT according to the manufacturer's instructions. Cells were washed twice in the respective Perm Buffer. Intracellular staining was done in the Permeabilization Buffer of the respective kit overnight (14-18 h at 4 °C). Cells were washed once in PBS/FCS and then resuspended in PBS/FCS containing 5% Precision Count Beads (Biolegend) for determination of cell number measured by the flow cytometer. Samples were acquired on a 5-laser spectral cytometer (Cytek Aurora, Cytek Biosciences) using spectral unmixing with autofluorescence extraction. Data were unmixed with SpectroFlo (Version 3.3) and analysed using FlowJo (Version 10, LLC).

Antibodies used for **surface staining and T cell phenotyping** were CD8α-SparkUV387 (Clone: 53-6.7, BioLegend), CD8α-BUV395 (Clone: 53-6.7, BD Biosciences), Bst2-BUV395 (Clone: Y129, BD Biosciences), TCRβ-BUV496 (Clone: H57-597, BD Biosciences), NK1.1-BUV563 (Clone: PK136, BD Biosciences), KLRG1-BUV661 (Clone: 2F1, BD Biosciences), CD4-BUV737 (Clone: RM4-5, BD Biosciences), CD45.1-BUV737 (Clone: A20, BD Biosciences), CD11c-BUV737 (Clone: N418, BD Biosciences), CD11c-BUV805 (Clone: N418, BD Biosciences), hCD2-BV421 (Clone: RPA-2.10, BioLegend), hCD2-PE (Clone: RPA-2.10, BioLegend), XCR1-BV421 (Clone: ZET, BioLegend), XCR1-BUV805 (Clone: ZET, BD Biosciences), TCR Vα2-BV421 (Clone: B20.1, BioLegend), PD-1-BV510 (Clone: 29F.1A12, BioLegend), CD90.2-BV570 (Clone: 30-H12, BioLegend), CD73-BV605 (Clone: TY/11.8, BioLegend), TCRδ-BV605 (Clone: GL3, BioLegend), CD69-BV650 (Clone: H1.2F3, BioLegend), CD69-BV785 (Clone: H1.2F3, BioLegend), Vsig4-BV605 (Clone: JAV4, BD Biosciences), CD44-BV711 (Clone: IM7, BioLegend), CD44-BV785 (Clone: IM7, BioLegend), TCR Vα2-FITC (Clone: B20.1, BioLegend), TCRδ-FITC (Clone: GL3, BioLegend), CD209α-FITC (Clone: MMD3, BioLegend), MHCII-SparkBlue550 (Clone: M5/114.15.2, BioLegend), MHCII-PE-Cy7 (Clone: M5/114.15.2, BioLegend), Nrp-1-RealBlue780 (Clone: V46-1954, BD Biosciences), CCR7-PE (Clone: 4B12, BioLegend), CCR7-PE-Cy5 (Clone: 4B12, BioLegend), CXCR6-PEDazzle594 (Clone: SA051D1, BioLegend), CD64-PEDazzle594 (Clone: W18349C, BioLegend), CD62L-PE-Cy5 (Clone: MEL-14, BioLegend), F4/80-PE-Cy5 (Clone: BM8, BioLegend), FR4-PE-Cy7 (Clone: 12A5, BioLegend), PD-1-PE-Cy7 (Clone: 29F.1A12, BioLegend), CXCR3-APC (Clone: CXCR3-173, BioLegend), CD45-R718 (Clone: 30-F11, BD Biosciences), CD45.2-AlexaFluor700 (Clone: 104, BioLegend), and CD4-APCFire810 (Clone: GK1.5, BioLegend).

Antibodies used for **nuclear staining** were Foxp3-eFluor450 (Clone: FJK-16s, Invitrogen), Foxp3-PE (Clone: FJK-16s, Invitrogen), Rorγt-BV480 (Clone: Q31-378, BioLegend), Ki-67-RealBlue705 (Clone: B56, BioLegend), Ki-67-FITC (Clone: B56, BioLegend), Ki-67-PE-efluor610 (Clone: SolA15, Invitrogen), and Helios-PE (Clone: 22F6, BioLegend). Antibodies used for **cytosolic staining** were IFNγ-PE (Clone: XMG1.2, BioLegend) and IL-17-AlexaFluor647 (Clone: TC11-18H10.1, BioLegend).

bioRxiv preprint doi: https://doi.org/10.64898/2026.01.13.698576; this version posted January 13, 2026. The copyright holder for this preprint (which was not certified by peer review) is the author/funder, who has granted bioRxiv a license to display the preprint in perpetuity. It is made available under aCC-BY 4.0 International license.

Sequential multiplex immunohistochemistry

Sequential multiplex immunohistochemistry (mIHC) was performed as previously described(*53, 54*) The antibody elution buffer was prepared by mixing 675 μL distilled water, 125 μL 0.5 M Tris-HCl pH 6.8, 200 μL 10% (w/v) sodium dodecyl sulfate, and 8 μL 2-mercaptoethanol. The following primary antibodies were used: anti-CD11c (clone D1V9Y, Cell Signaling, 1:500), anti-CD3e (clone E4T1B, Cell Signaling, 1:400), anti-CD4 (clone 4SM95, Thermo Fisher, 1:400), anti-CD8 (clone 4SM15, Thermo Fisher, 1:400), anti-Clec4f (clone 370901, R&D Systems, 1:1000), anti-Foxp3 (clone FJK-16s, Thermo Fisher, 1:200), anti-IBA1 (polyclonal, VWR #100369-764, 1:1000), anti-Lyve-1 (polyclonal, Abcam #ab14917, 1:200), anti-MHC-II (clone M5/114.15.2, BioLegend, 1:200), anti-PD-1 (polyclonal, R&D Systems #AF1021, 1:200), and anti-PD-L1 (clone E1L3N, Cell Signaling, 1:200).

Secondary antibodies included Alexa Fluor® 647–conjugated anti-rabbit IgG (H+L), F(ab')$_2$ fragment (Cell Signaling #4414, 1:1000), Alexa Fluor® 750–conjugated goat anti-rat IgG H&L (Abcam #ab175751, 1:500), Alexa Fluor™ 750–conjugated goat anti-rabbit IgG (H+L), cross-adsorbed (Invitrogen #A-21039, 1:500), and Alexa Fluor® 647–conjugated anti-rat IgG (H+L) (Cell Signaling #4418, 1:1000).

Image analysis was performed with an in-house optimized image processing pipeline and software tools.(*53*) Clusters were defined as consisting of at least 3 cells of each cell type (CD3+ or CD11c+IBA1-Clec4f-) clustering in direct proximity and counted manually across the whole slides.

Immunofluorescence imaging of CCR7KO and WT mice (Ext, Data 7a):

Samples were fixed in 4% PFA, embedded and cryosectioned. Samples were blocked with serum and stained with aCD3 (500A2, hamster) – FITC 1:200 and 1:1000 DAPI overnight, followed by a wash step in >5ml wash/stain buffer for >4h at 4°C and constant agitation and mounted on glass slides with ProLongGold.After imaging, CD3+ cells were counted manually and in a blinded manner by 3 different persons. The mean of the results of the three counting persons was plotted for each slide.

scRNASeq experiment and analysis

Liver cells were isolated as detailed in the isolation section. All pipetting steps were performed with wide-orifice low-binding pipets (VWR), After collecting the pellet from the Tissue Grinder device (FFX Technologies), cells were resuspended in 50 mL PBS/FCS and hepatocytes were pelleted by centrifugation at 50 x g for 2 min at 4 °C. The supernatant was centrifuged for 8 min at 400 x g at 4 °C to pellet the fraction enriched for immune cells. The pellet was resuspended in PBS/1% BSA, cells were counted and stained for 30 min with the corresponding TotalSeqC-Hashtag antibody 1-3 (Biolegend) at 4 °C at a dilution of 1:3200 in 50 μL PBS/1% BSA per million cells.After incubation, 5 mL of PBS/0.04% BSA were added to the cell suspension and cells were pelleted by centrifugation for 5 min at 400 x g. The pellet was resuspended in 5 mL of PBS, incubated for 5 min at RT, then transferred to a new tube, centrifuged down and washed twice PBS/1%BSA. Cells from the three replicates were then pooled and counted again. TotalSeqC-antibodies (Biolegend) for protein detection (TotalSeq-C1058, C0077, C0073, C0004; BioLegend) and flow cytometry staining antibodies (CD4-

bioRxiv preprint doi: https://doi.org/10.64898/2026.01.13.698576; this version posted January 13, 2026. The copyright holder for this preprint (which was not certified by peer review) is the author/funder, who has granted bioRxiv a license to display the preprint in perpetuity. It is made available under aCC-BY 4.0 International license.

PECy7, Clone: GK1.5, BioLegend, TCRb-APC, Clone: H57-597, Biolegend, CD45-FITC, Clone: 30-F11, Biolegend) were added in their respective dilutions and stained for 30 min at 4 °C. After incubation, 5 mL of PBS/0.04% BSA were added to the cell suspension and cells were pelleted by centrifugation for 5 min at 400 x g. The pellet was resuspended in 5 mL of PBS, incubated for 5 min at RT, then transferred to a new tube, centrifuged down and washed once with PBS/1%BSA. DAPI was added for detection of dead cells and incubated for 2 min at 4 °C. Then, cells were centrifuged down for 5 min at 400 x g and resuspended in PBS/FCS for FACS sorting. Cells were sorted at the Aria II or Aria Fusion sorter (BD Biosciences) into 1.5 mL tubes with RPMI/10% FCS at 4°C to collect at least 20.000 CD4 T cells and 200.000 other immune cells. Cells were then washed with PBS/FCS, counted again and adjusted to a concentration of 1.000 cells/µL. CD4 T cells and other immune cells were pooled in ratio of 1 + 3 aiming for a cell recovery of 2.500 CD4 T cells and 7.500 other immune cells per sample. Then, cells were put on the Next GEM Chip K to initiate the library generation workflow (10X Genomics). Single cell RNA sequencing workflow was performed with the 5` Immune Profiling with Feature Barcoding Kit (10X Genomics) according to the manufacturer`s instructionsLibraries were quality controlled at the TapeStation (Agilent) sequenced on the Illumina NovaSeq system with an SP (for shallow sequencing to determine library composition) or S1 cartridge and preprocessed via the Cell Ranger pipeline by the Genomic Facility at IZKF Aachen.

Raw FASTQ data was analysed with the “CellRanger” pipeline (10X Genomics) and then in the R package Seurat(*55*) (v5.0.3.) to process all data: To demultiplex the biological replicates by hashtag antibodies, generate a separate data assay for hashtag antibodies along with the transcriptomics assay, we applied the function “HTODemux”. Benchmarking several options for “quantile” yielded the cutoff 0.99 for an optimal separation of cells per mouse. Cells assigned to more than 1 hashtag were classified as doublets and removed from the dataset. Cells predicted to be negative for hashtags were only removed for analyses of individual mice, e.g. in clonotype analysis, but left in the data for reclustering and DE gene analysis. Ambient RNA contamination was predicted by “decontX” from the “celda” package(*56*) (v1.18.2). Few remaining potential doublets were predicted and removed by the package DoubletFinder(*57*)(v2.0.4). For quality control, cells containing more than 5 % mitochondrial RNA counts, with a complexity (log10(nFeature_RNA) / log10(nCount_RNA)) < 0.8 or an ncount of > 2.5 * IQR above the 75th quantile of the whole sample were removed.
Cell cycle scores for all cells were obtained by the built-in function “CellCycleScore” in Seurat and regressed out, as well as TCR genes (Trav and Traj genes), meaning they were not used for any clustering processes, while remaining in the data for DE gene comparisons. PCA and UMAP dimensional reduction were applied and clustering was performed using the built-in function RunPCA, RunUMAP, FindNeighbors and FindCluster in Seurat with a cluster resolution of 0.5. Data from different samples were integrated using the CCA method built-in Seurat. DE genes between clusters were found by conducting a Wilcoxon test with Benjamin-Hochberg correction. Ligand receptor analysis was performed by CellChatDB(v2.1.2, PMID: 39289562). Fig.s were prepared using Seurat and ggplot2 (v3.5.2).
To recluster only CD4 T cells, first all clusters containing T cells/ILCs and NK cells from Fig. S3c were reclustered (Fig. S3d) and then only CD4+ T cell containing clusters were reclustered to obtain the CD4 T cell UMAP (Fig. S3e) and merged into clusters comparable to flow analysis, using the following gene modules: naïve (Ccr7, Sell, Tcf7), Treg (Foxp3, Ikzf2, Ctla4), activated Tconv (Cxcr3, Id2, Cd44, Mki67) and NKT (Klrb1c)) These clusters were then used

bioRxiv preprint doi: https://doi.org/10.64898/2026.01.13.698576; this version posted January 13, 2026. The copyright holder for this preprint (which was not certified by peer review) is the author/funder, who has granted bioRxiv a license to display the preprint in perpetuity. It is made available under aCC-BY 4.0 International license.

for DE gene analysis. Heatmaps were generated by normalizing gene expression per gene from the Seurat object for each mouse.

For VDJ analysis, individual mice were separated by hashtag and the package SC repertoire was used. R20 and R50 values were calculated according to Connors et al (add ref). To recluster only DC, only myeloid cell clusters were chosen from the UMAP in Fig. S7a, reclustered, manually annotated according to cluster marker and canonical genes and only DC containing clusters were chosen. Clusters were merged and a T cell contamination was removed (Fig. S6f), resulting in the UMAP shown in Fig. 3c.

Raw FASTQ data has been deposited in ENA with accession code PRJEB104713 and under the Zenodo ID (DOI:10.5281/17940609

Xenium spatial transcriptomics

For 10X Xenium targeted spatial transcriptomics, we designed a 480-plex custom panel based on our scRNASeq data and the Liver Cell Atlas[56]. Sample processing was done on 5 µm thick paraffine embedded liver sections as described by the manufacturer using the Cell Segmentation kit and Xenium V1 chemistry. Analysis of the data was conducted using the SPARROW pipeline(*58*). The main steps consisted of image pre-processing, nuclei segmentation, transcript registration based on the new segmentation, cell type annotation, and cell neighbourhood analysis.

Image pre-processing was performed on the DAPI channel with the aim to improve the segmentation results. We applied a min-max filter with a size of 35 pixels to subtract background noise, and contrast enhancement using the CLAHE algorithm with a clip limit of 3.5. Nuclei segmentation was then performed on the pre-processed image using the cellpose model(*59*), which is a U-Net-like convolutional neural network (depth of 50 pixels and reflective boundary for the mapping overlap). The cellpose model was applied using an estimated nucleus diameter of 7 pixels; flow error threshold of <0.65, minimum area of 50 total pixels for segmented objects; probability threshold of >0.0; size fraction <0.4 0.4. The parameters for both the image pre-processing and segmentation were chosen based on grid searches and visual examination of the results. A combination of 25 different parameters for pre-processing and 156 for the segmentation were scanned.

Transcript registration was done using the new nuclei segmentation layer and the transcriptomics data was normalised based on the nuclei sizes. We removed the *Isg15*, *Top2a, Birc5, Ube2c, Cenpa* and *Mki67* genes for the downstream analysis, as these genes were strongly driving the clustering.

Leiden clustering was performed using the default parameters(*60*). We looked at the top 20 genes of each cluster and annotated DCs and T cells the clusters base on expression of canonical marker genes. Extended Data 7b displays the results of the nuclei segmentation highlighting the DCs and T cells that were identified using Leiden clustering. In order to formally define DC-T cell clusters, we applied the DBScan algorithm implemented by the scikit-learn Python library (v1.7.1) using the centroid coordinates of the annotated DCs and T cells (Fig. S7c). DE gene analysis was conducted for DCs and T cells in and outside

bioRxiv preprint doi: https://doi.org/10.64898/2026.01.13.698576; this version posted January 13, 2026. The copyright holder for this preprint (which was not certified by peer review) is the author/funder, who has granted bioRxiv a license to display the preprint in perpetuity. It is made available under aCC-BY 4.0 International license.

clusters (Figs. S7f&i): Volcano plots display the results marking the top 10 DE genes. As close proximity of the cells leads to detection of transcripts from neighbouring cells (e.g. MHCII genes in T cells and CD3 in DCs) even with our very conservative nuclei segmentation, we filtered the DE labelled genes for T cell/DC genes from our panel only for Fig. 3j.

The raw and processed 10X Xenium data are available in Zenodo, ID17940609:

AI-assisted manuscript preparation

ChatGPT (OpenAI; model: GPT-4.5/5.1 Thinking) was used during manuscript preparation to assist with language editing (clarity, concision, and organization) and to generate alternative phrasing. The tool was not used to generate or analyze primary data, to perform statistical analyses, or to generate references/citations. All AI-assisted text was reviewed, edited, and verified by the authors.

bioRxiv preprint doi: https://doi.org/10.64898/2026.01.13.698576; this version posted January 13, 2026. The copyright holder for this preprint (which was not certified by peer review) is the author/funder, who has granted bioRxiv a license to display the preprint in perpetuity. It is made available under aCC-BY 4.0 International license.

Supplementary figure 1

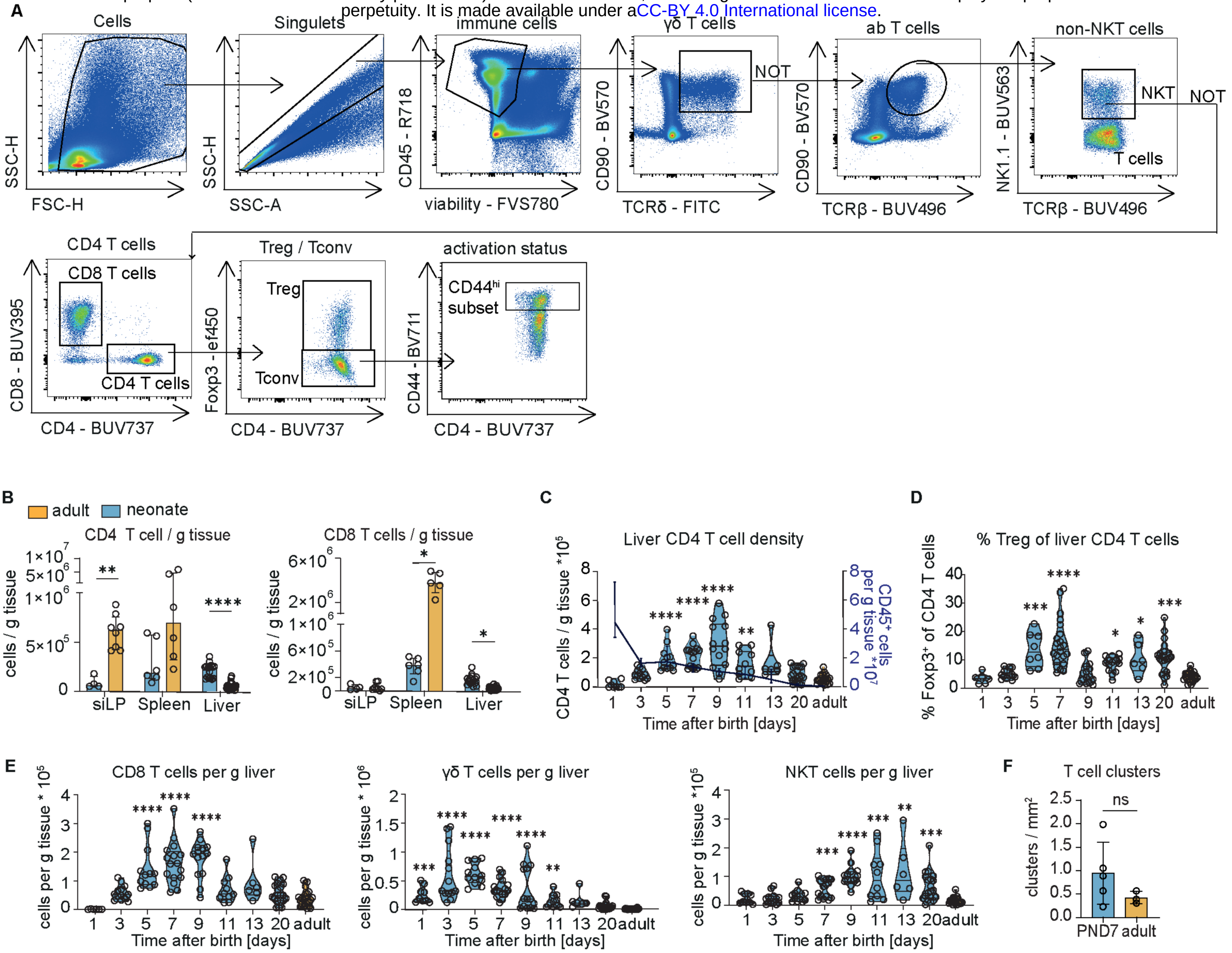

Supplementary figure 1: Flow cytometric quantification of T cells in the neonatal liver.

(A) Gating strategy for the identification of T cell subsets and phenotype used in all flow cytometry experiments (if not indicated otherwise).

(B) Cell densities of CD4 and CD8 T cells in liver tissue in small intestinal lamina propria (siLP), Spleen and liver. Multiple Mann Whitney U test. 4-10 mice from 1-2 independent experiments.

(C) Kinetics of CD4 T cells (violin plots) and total CD45⁺ cell density (line showing median + IQR) in the neonatal liver. Kruskal Wallis test + Dunn`s comparison of each time point to adult group. Violin plots of 6-20 mice per timepoint from 2-6 independent experiments.

(D) % of Tregs (Foxp3⁺) within CD4 T cells in the neonatal liver. Kruskal Wallis test + Dunn`s comparison of each time point to adult group. Violin plots of 6-20 mice per timepoint from 2-6 independent experiments.

(E) Kinetics of CD8 T, γδT and NKT cell density in the neonatal liver. Kruskal Wallis test + Dunn`s comparison of each time point to adult group. Violin plots of 6-20 mice per timepoint from 2-6 independent experiments.

(F) Ext. Data 1f: Density of manually counted microclusters of CD3⁺ cells per slide in neonatal (7-day-old) and adult WT livers (3-5 mice per group). Unpaired t-test.

Asterisks indicate significance levels: * < 0.05, ** < 0.01, *** < 0.001 **** < 0.0001. All tests were performed in a two-sided manner.

bioRxiv preprint doi: https://doi.org/10.64898/2026.01.13.698576; this version posted January 13, 2026. The copyright holder for this preprint (which was not certified by peer review) is the author/funder, who has granted bioRxiv a license to display the preprint in perpetuity. It is made available under aCC-BY 4.0 International license.

Supplementary figure 2

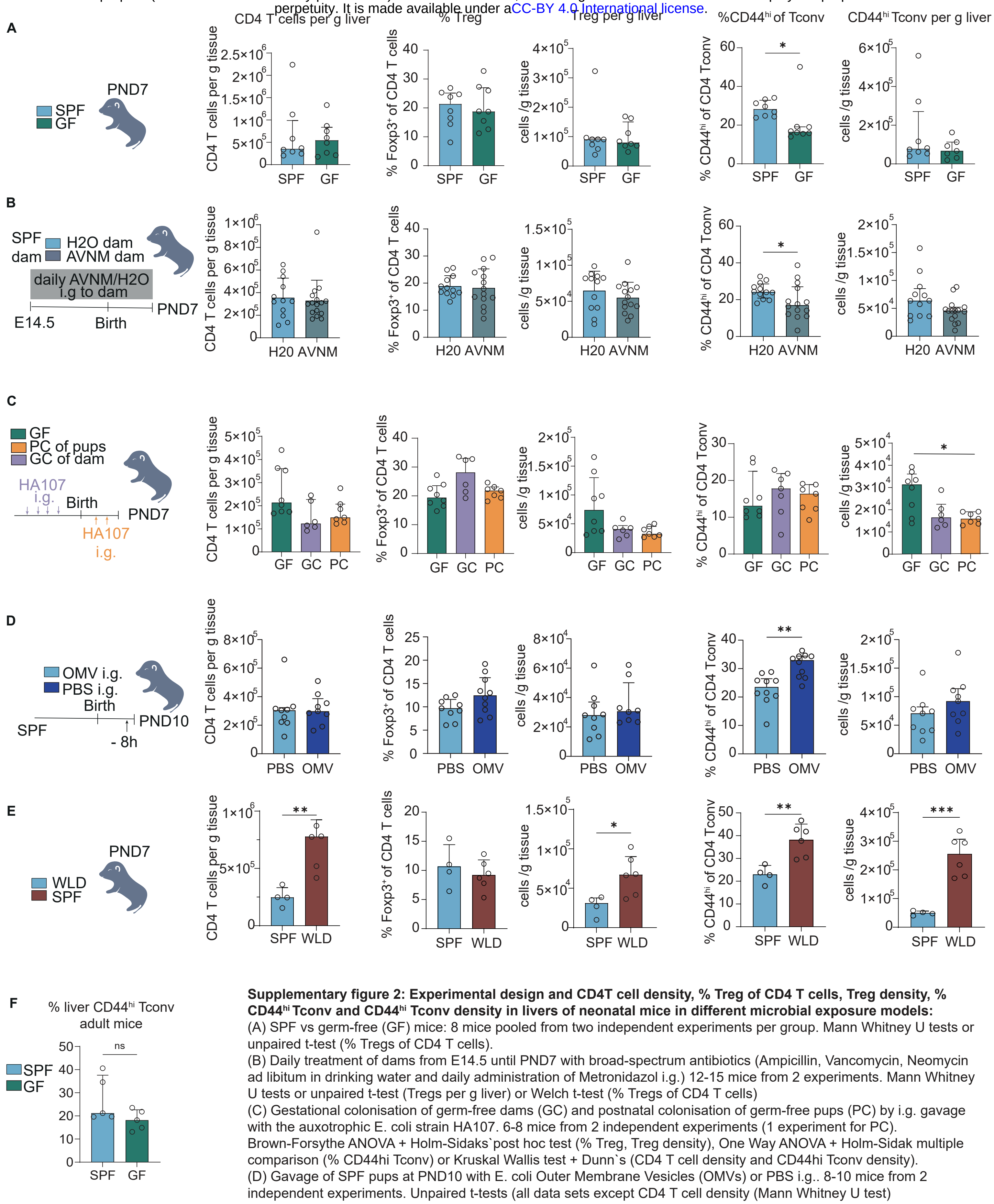

Supplementary figure 2: Experimental design and CD4T cell density, % Treg of CD4 T cells, Treg density, % CD44^hi Tconv and CD44^hi Tconv density in livers of neonatal mice in different microbial exposure models:
(A) SPF vs germ-free (GF) mice: 8 mice pooled from two independent experiments per group. Mann Whitney U tests or unpaired t-test (% Tregs of CD4 T cells).
(B) Daily treatment of dams from E14.5 until PND7 with broad-spectrum antibiotics (Ampicillin, Vancomycin, Neomycin ad libitum in drinking water and daily administration of Metronidazol i.g.) 12-15 mice from 2 experiments. Mann Whitney U tests or unpaired t-test (Tregs per g liver) or Welch t-test (% Tregs of CD4 T cells)
(C) Gestational colonisation of germ-free dams (GC) and postnatal colonisation of germ-free pups (PC) by i.g. gavage with the auxotrophic E. coli strain HA107. 6-8 mice from 2 independent experiments (1 experiment for PC). Brown-Forsythe ANOVA + Holm-Sidaks`post hoc test (% Treg, Treg density), One Way ANOVA + Holm-Sidak multiple comparison (% CD44hi Tconv) or Kruskal Wallis test + Dunn`s (CD4 T cell density and CD44hi Tconv density).
(D) Gavage of SPF pups at PND10 with E. coli Outer Membrane Vesicles (OMVs) or PBS i.g.. 8-10 mice from 2 independent experiments. Unpaired t-tests (all data sets except CD4 T cell density (Mann Whitney U test)
(E) SPF vs wildlings: 4-6 per group mice from 1 experiment. Unpaired-tests (CD4 T cell density, Treg density, % Treg) or Welch t-tests (Cd44^hi % and density)
(F) % CD44^hi of Tconvs in livers of 8-10 week old SPF and germ-free mice (GF). 5 mice per group from 2 independent experiments. Unpaired t-test.
All experiments containing flow cytometry were done with the compared groups within 1 experiment processed on the same day. Asterisks indicate significance levels: * < 0.05, ** < 0.01, *** < 0.001 **** < 0.0001. All tests were performed in a two-sided manner.

bioRxiv preprint doi: https://doi.org/10.64898/2026.01.13.698576; this version posted January 13, 2026. The copyright holder for this preprint (which was not certified by peer review) is the author/funder, who has granted bioRxiv a license to display the preprint in perpetuity. It is made available under aCC-BY 4.0 International license.

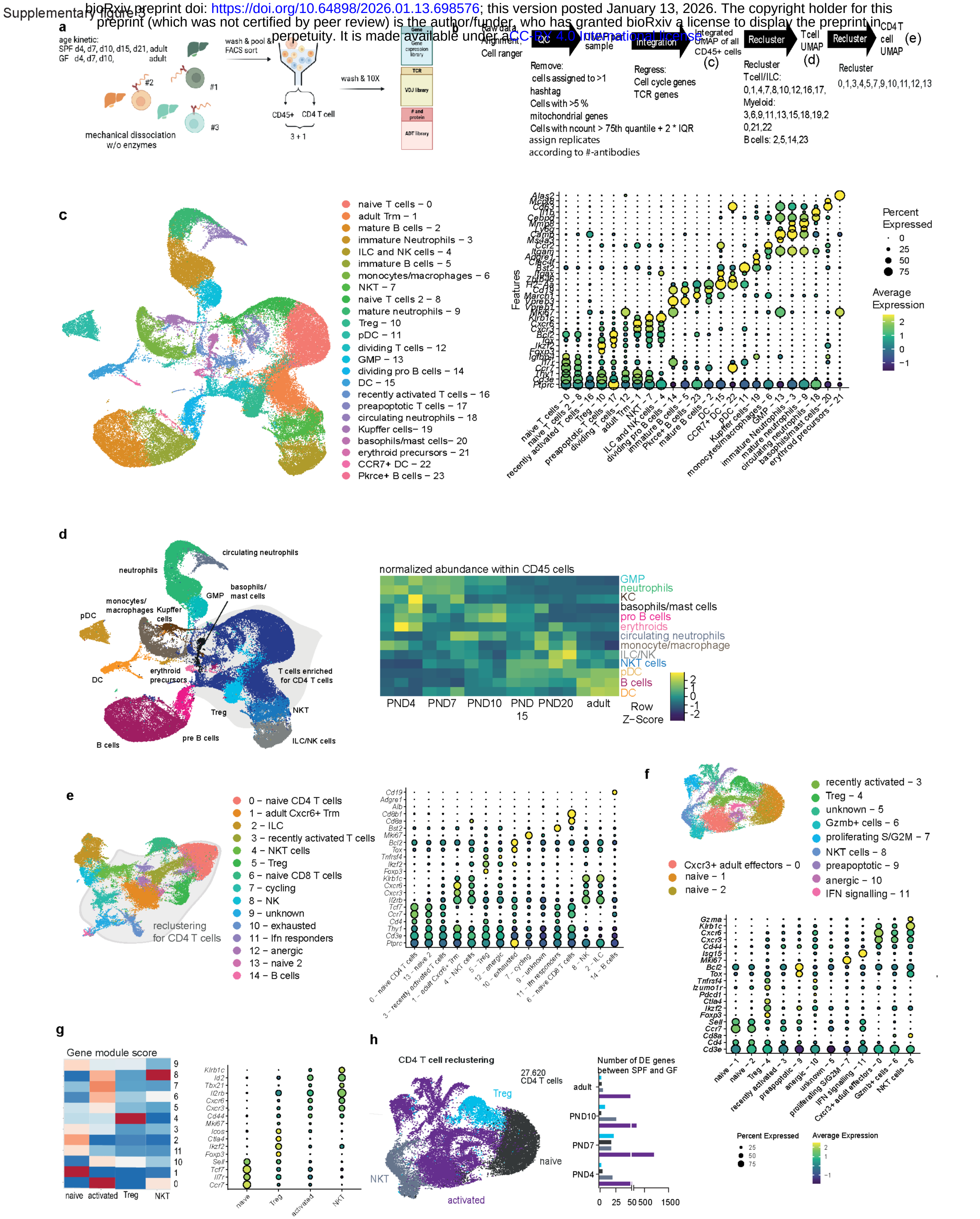

Supplementary figure 3: Analysis strategy of scRNASeq data and Extended Data.
(A) Experimental design of scRNASeq experiment with TotalSeqC- hashtagged biological replicates (1 mouse per hashtag) and enrichment for CD4 T cells. Graphic created with Biorender.
(B) Flow chart detailing QC metrics and reclustering strategy for scRNASeq data.
(C) UMAP of all CD45+ cells, bubbleplot with canonical marker genes used for annotation; this UMAP was used for Cell Chat analysis in Figure 3.
(D) UMAP of all CD45+ cells with merged clusters and heatmap of the z-score normalized % of total sample for each SPF mouse over the kinetic.
(E) Reclustered UMAP of all T cells and ILCs and bubbleplot with canonical marker genes used for annotation.
(F) Reclustered UMAP of CD4 T cells and bubbleplot with canonical marker genes used for annotation.
(G) Gene modules and gene module scores of the clusters from (f) for merging of the clusters: naive: Ccr7, Sell, Tcf7; activated: CD44, Mki67, Id2; Treg: Foxp3, Ikzf2, Ctla4; NKT: Klrb1c
(H) Merged CD4 T cell UMAP and number of DE genes in each cluster between SPF and GF mice at different time points after birth with bar plots color-coded by cluster.

bioRxiv preprint doi: https://doi.org/10.64898/2026.01.13.698576; this version posted January 13, 2026. The copyright holder for this preprint (which was not certified by peer review) is the author/funder, who has granted bioRxiv a license to display the preprint in perpetuity. It is made available under aCC-BY 4.0 International license.

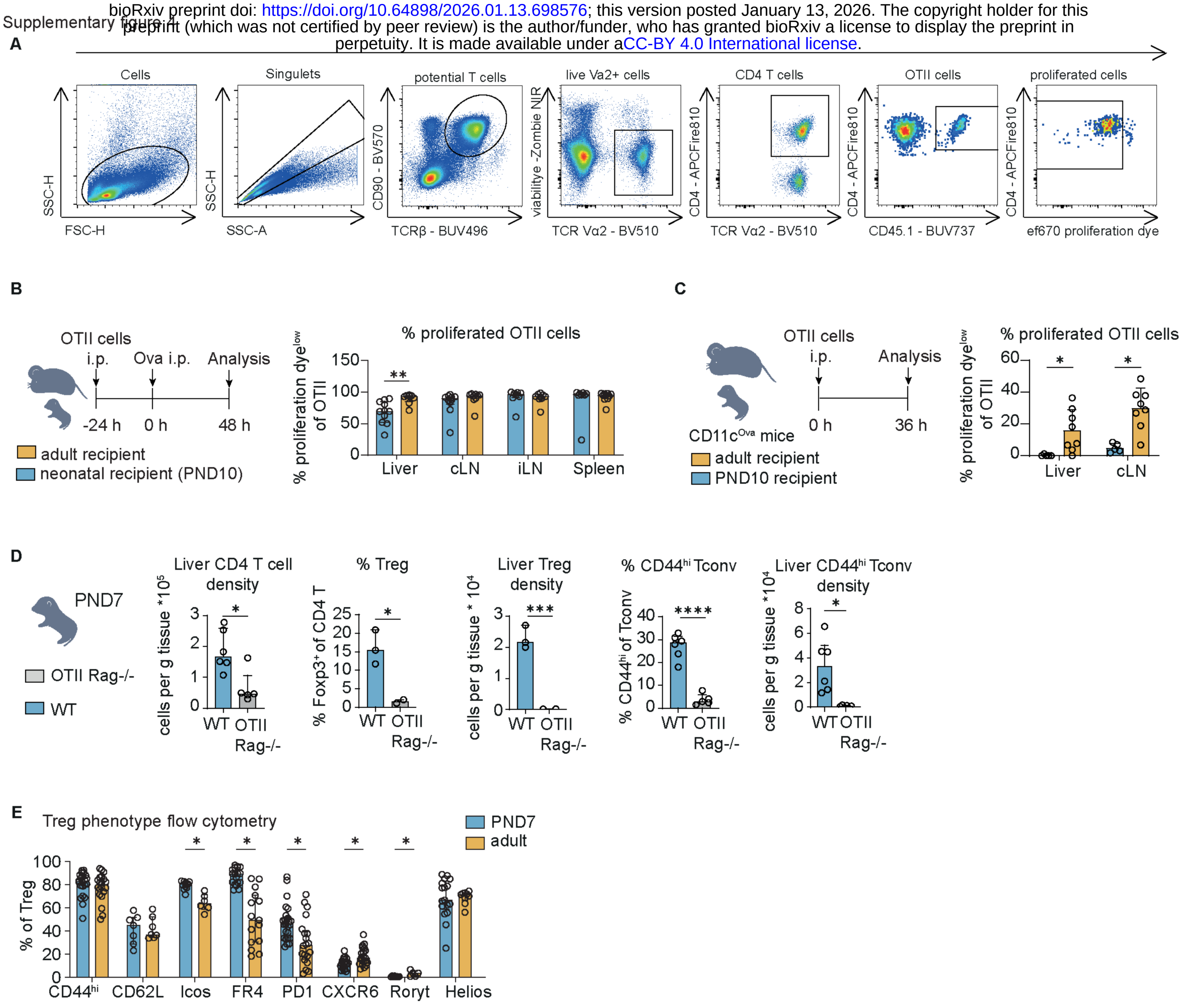

Supplementary figure 4: OTII experiments show delayed proliferation in neonatal liver tissue.

(A) Gating strategy for identification of proliferated OTII cells for (b) and (c)

(B) Experimental design of OTII transfer and intraperitoneal ovalbumin (ova) injection into neonatal and adult WT mice and % of proliferated cells of OTII cells in liver, celiac LN (cLN), inguinal LN (iLN) and spleen 48 h after antigen exposure. 8-10 mice from 2 independent experiments. Multiple Mann Whitney U test. + Holm-Sidak multiple test correction.

(C) Experimental design of OTII transfer into neonatal and adult CD11c-Ova mice and % proliferated cells of OTII cells in liver and celiac LN (cLN) 48 h after antigen exposure. 7-9 mice from from 2 independent experiments. Multiple Mann Whitney U test + Holm-Sidak multiple test correction.

(D) CD4 T cell density, Treg frequency and density, and activated Tconv frequency and density in the liver 7-day old B6 WT SPF or OTII RAG−/− mice (no exposure to the OTII antigen Ova in either of the groups. Barplots show median and IQR of 2 pooled experiments (1 experiment for Treg data) with 2-6 pups per group. Welch t-tests.

(E) Treg phenotypic marker expression in PND7 and adult livers measured by flow cytometry. 6-19 mice per group from 3-5 independent experiments. Multiple Mann Whitney U test.

All experiments containing flow cytometry were done with the compared groups within 1 experiment processed on the same day. Data show median and IQR if not otherwise indicated. Asterisks indicate significance levels: * < 0.05, ** < 0.01, *** < 0.001 **** < 0.0001 or not significant if there is no asterisk. All tests were performed in a two-sided manner.

bioRxiv preprint doi: https://doi.org/10.64898/2026.01.13.698576; this version posted January 13, 2026. The copyright holder for this preprint (which was not certified by peer review) is the author/funder, who has granted bioRxiv a license to display the preprint in perpetuity. It is made available under aCC-BY 4.0 International license.

Supplementary figure 5

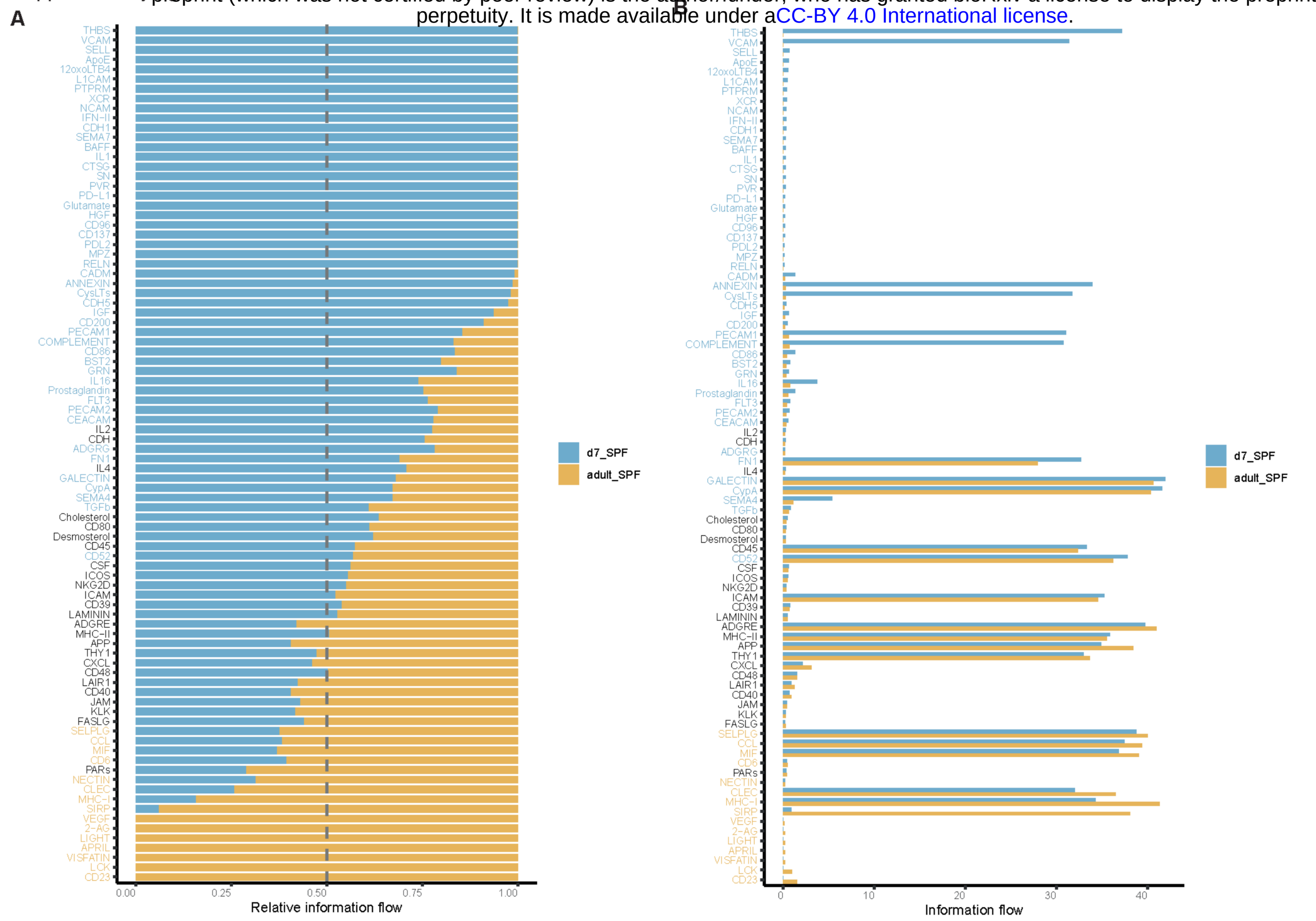

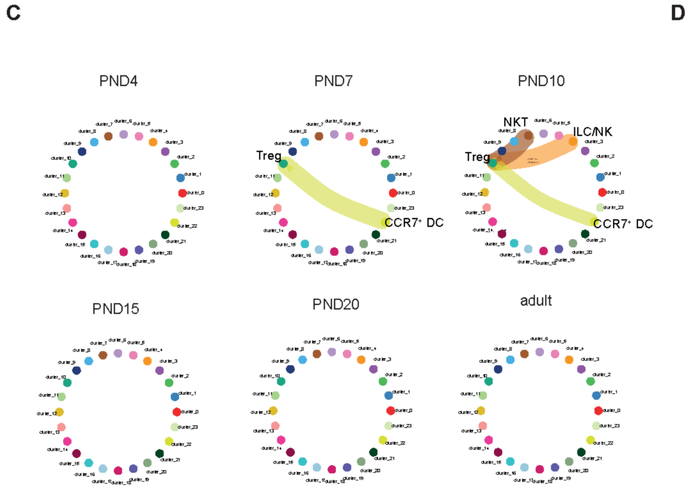

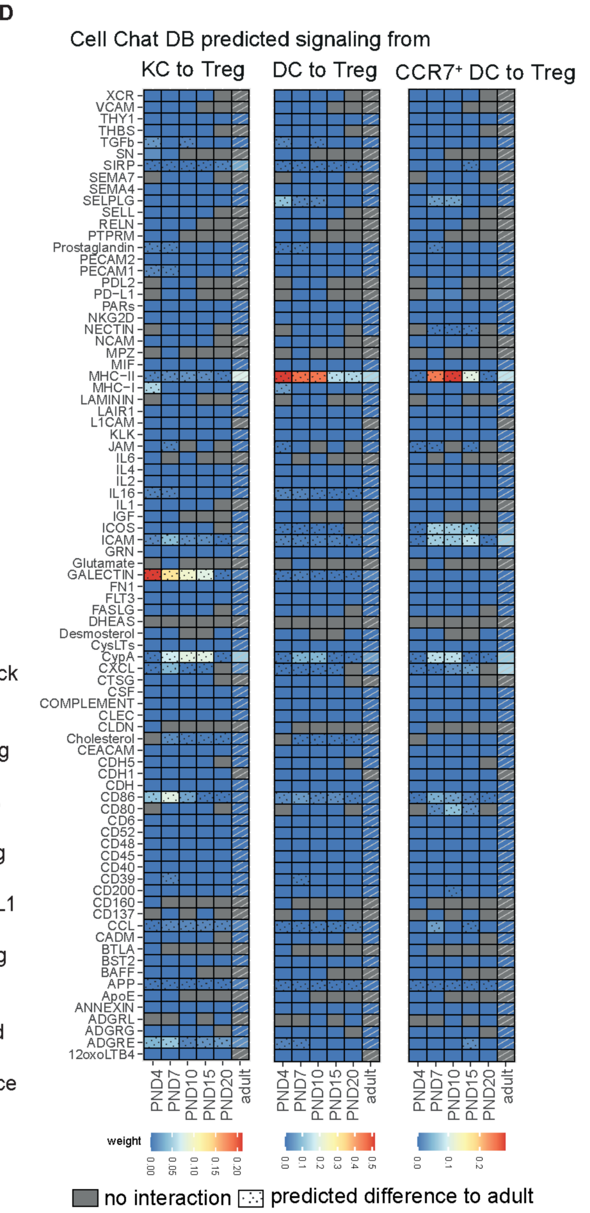

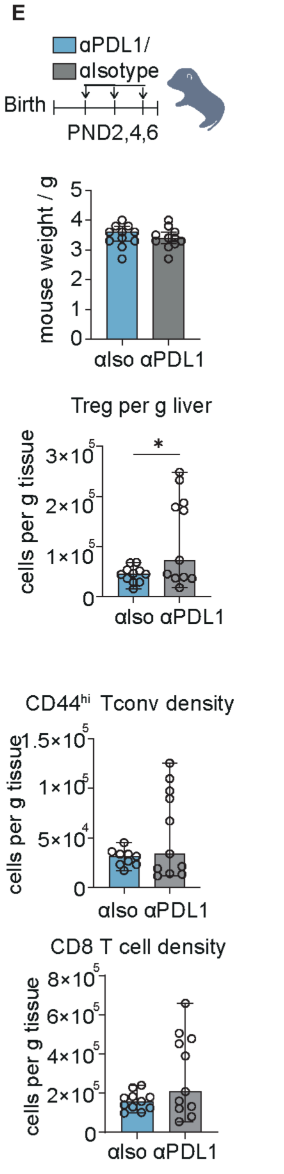

Supplementary figure 5: Cell Chat analysis and PDL1-blockade

(A) Comparison of relative information flow between neonatal (PND7) and adult liver total immune cells predicted by Cell Chat DB (blue = enriched signalling in neonate, yellow = enriched signalling in adult; black = no difference).

(B) Absolute information flow between neonatal (PND7) and adult liver total immune cells predicted by Cell Chat DB. (blue = enriched signalling in neonate, yellow = enriched signalling in adult; black = no difference)

(C) Circle plots indicating PD-L2 signaling in SPF at different timepoints after birth. Cluster indicate the clusters from Extended data 3c.

(D) Heatmaps displaying all CellChat DB pathways signaling to the Treg cluster from either KC, DC or CCR7⁺ DC.

(E) Experimental design for neonatal PD-L1 blockade, weights of αPD-L1 treated neonates and their littermates treated with αIsotype antibody a PND7, Tregs per g liver, CD44^{hi} Tconvs per g liver and CD8 T cells per g liver. Unpaired t-test (mouse weight) and Mann Whitney U tests. 9-11 pups from 3 independent experiments.

All experiments containing flow cytometry were done with the compared groups within 1 experiment processed on the same day. Data show median and IQR if not otherwise indicated. Asterisks indicate significance levels: * < 0.05, ** < 0.01, *** < 0.001 **** < 0.0001 or not significant if there is no asterisk. All tests were performed in a two-sided manner.

bioRxiv preprint doi: https://doi.org/10.64898/2026.01.13.698576; this version posted January 13, 2026. The copyright holder for this preprint (which was not certified by peer review) is the author/funder, who has granted bioRxiv a license to display the preprint in perpetuity. It is made available under aCC-BY 4.0 International license.

Supplementary figure 6:

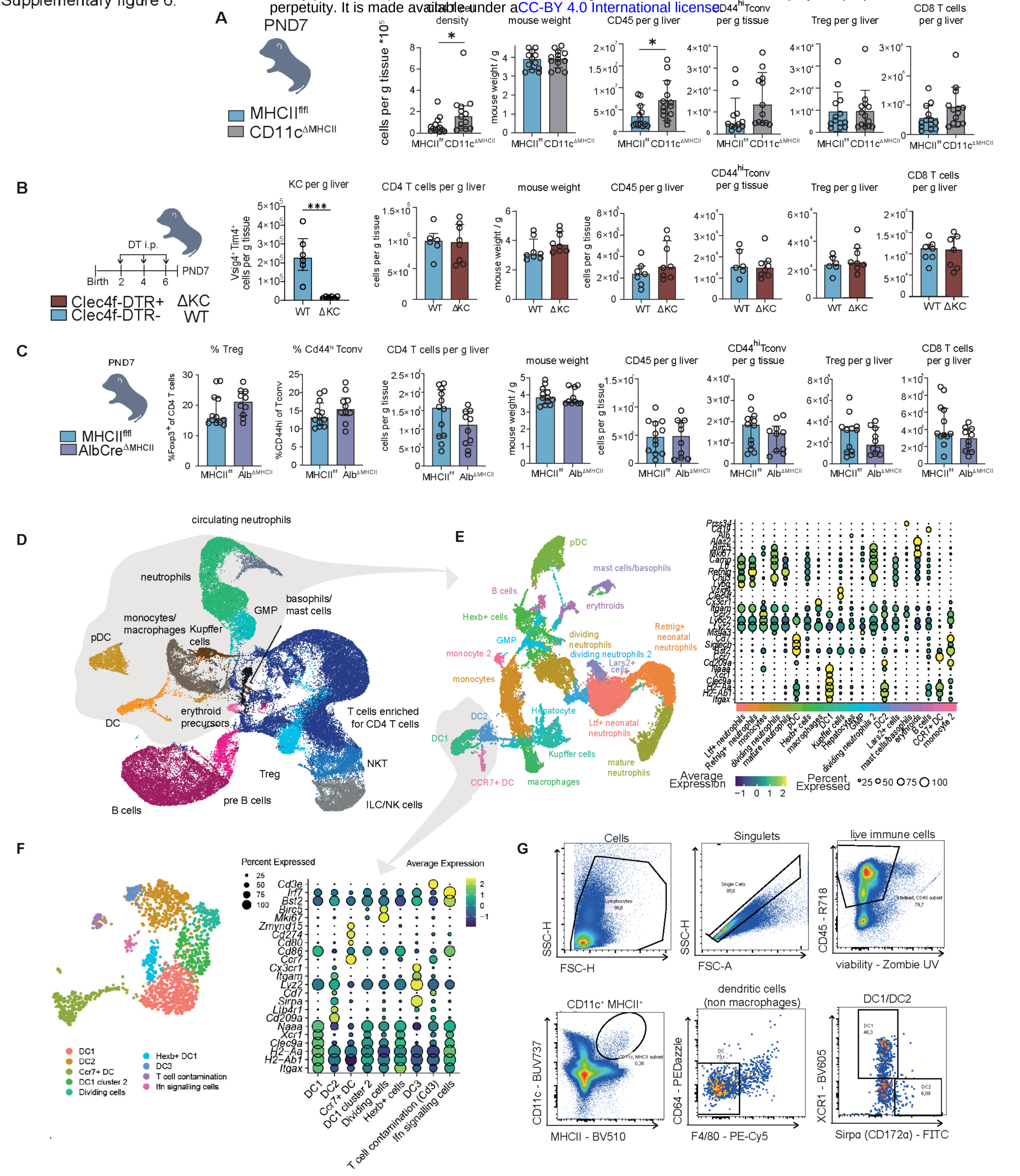

Supplementary figure 6: Flow cytometry of neonatal mice with conditional MHCII knock-out or APC depletion.

(A) Extended flow cytometry data for neonatal CD11cCre MHCIIflfl mice and their Cre- littermates. Liver CD4 T cell density, mouse weights at PND7, CD45+ cell per g liver, CD44hi Tconvs per g liver, Tregs per g liver and CD8 T cells per g liver. 9-12 mice per group pooled from 4 independent experiments. Mann Whitney U tests.

(B) Extended flow cytometry data for neonatal depletion of Kupffer cells. Clec4f-DTR+ and Celec4f-DTR- littermates; KC density in liver tissue (Welch t-test); liver CD4 T cell density, mouse weights at PND7, CD45+ cell per g liver, CD44hi Tconvs per g liver, Tregs per g liver and CD8 T cells per g liver (unpaired t-tests). 6-8 mice per group pooled from 2 independent experiments.

(C) Liver CD4 T cell phenotypes in neonatal AlbCre MHCIIflfl mice and their Cre negative littermates. 9-12 mice per group from 3 independent experiments. Mann Whitney U tests. Mann Whitney U tests.

(D) CD45+ UMAP used for reclustering of all myeloid cells (gray circle).

(E) Resulting Myeloid UMAP used for reclustering of DC (gray circle) and bubbleplot showing a selection of genes used for annotation.

(F) DC UMAP used for analysis and bubble plot bubbleplot showing a selection of genes used for annotation.

(G) Gating strategy for flow cytometry analysis of liver DC in neonatal and adult mice.

All experiments containing flow cytometry were done with the compared groups within 1 experiment processed on the same day. Data show median and IQR if not otherwise indicated. Asterisks indicate significance levels: * < 0.05, ** < 0.01, *** < 0.001 **** < 0.0001 or not significant if there is no asterisk. All tests were performed in a two-sided manner.

bioRxiv preprint doi: https://doi.org/10.64898/2026.01.13.698576; this version posted January 13, 2026. The copyright holder for this preprint (which was not certified by peer review) is the author/funder, who has granted bioRxiv a license to display the preprint in perpetuity. It is made available under aCC-BY 4.0 International license.

Supplementary figure 7

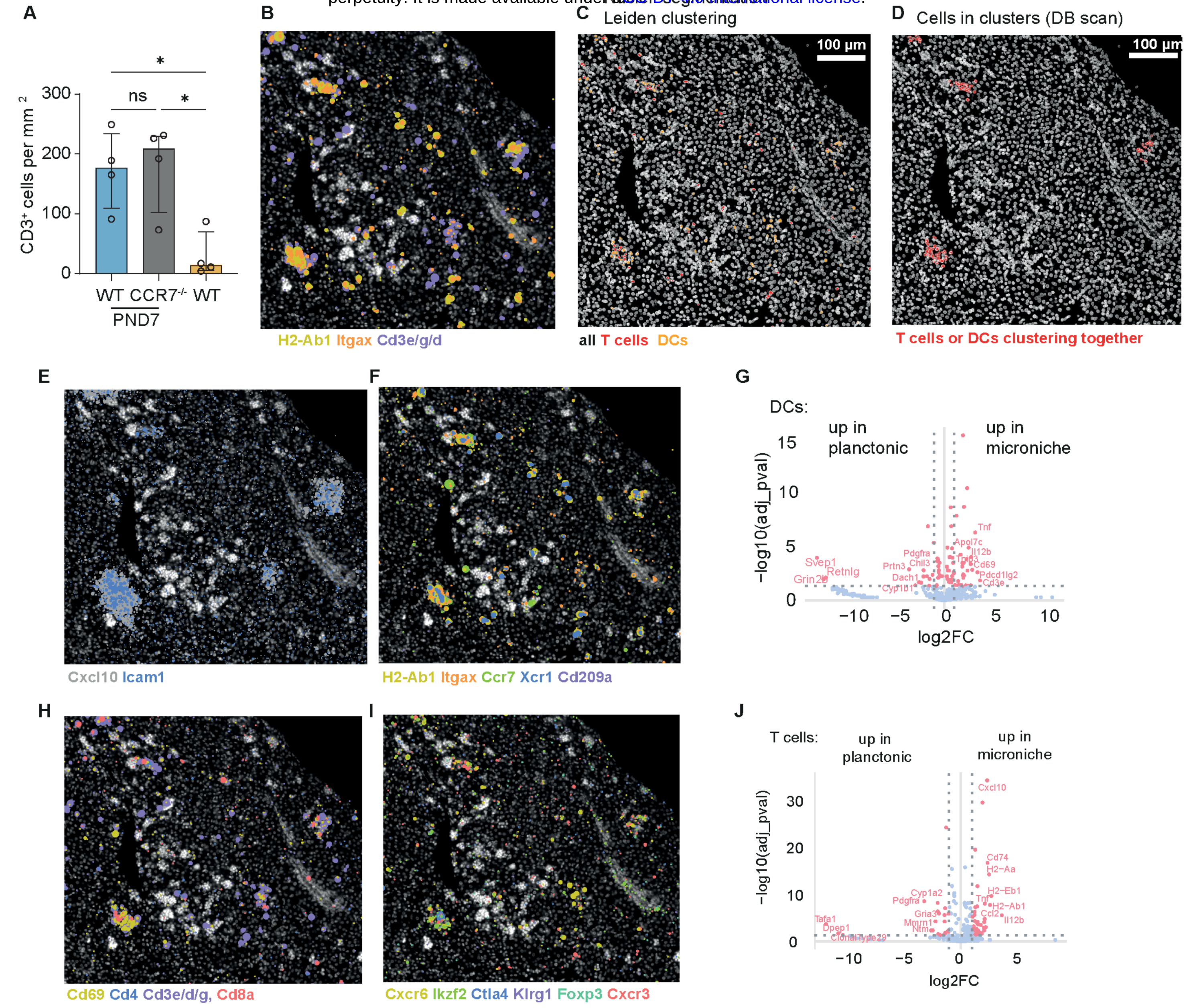

Supplementary figure 7: Ext. Data for Xenium spatial transcriptomics of neonatal liver.
(A) CD3+ cells per mm² in neonatal WT, CCR7 KO and adult WT mice, means of cell number manually counted in immunofluorescence microscopy pictures by 3 different persons in a blinded manner. Barplots show median + IQR of 4 mice per group. One Way ANOVA + Holm-Sidak`s multiple comparison.
(B) Overview DAPI image + expression of DC (*Itgax, H2-Ab1*) and T cell transcripts (*Cd3e, CD3g, CD3d*) in 7-day old liver.
(C) Segmented image with all DCs and T cells highlighted
(D) Segmented image showing DCs and T cells within clusters defined using DBScan.
(E) DAPI image + expression of *Cxcl10* and *Icam1* transcripts.
(F) DAPI image + expression of DC (*Itgax, H2-Ab1)* and subsets (DC1: *Xcr1*, DC2: *Sirpa*, CCR7+ DC: *Ccr7*)
(G) Volcano plot showing DE gene analysis of DCs inside and outside of microniches from Xenium panel. The top DE genes from the total panel are highlighted with labels.
(H) DAPI image + expression of T cell marker transcripts (*Cd69* (activation), *Cd4, Cd3e, Cd3g, Cd3d, Cd8a*)
(I) DAPI image + expression of Treg (*Foxp3, Ikzf2, Ctla4, Klrg1*) and tissue residency-associated transcripts (*Cxcr6, Cxcr3)*
(J) Volcano plot showing DE gene analysis of T cells inside and outside of microniches from Xenium panel. The top DE genes from the total panel are highlighted with labels.

bioRxiv preprint doi: https://doi.org/10.64898/2026.01.13.698576; this version posted January 13, 2026. The copyright holder for this preprint (which was not certified by peer review) is the author/funder, who has granted bioRxiv a license to display the preprint in perpetuity. It is made available under aCC-BY 4.0 International license.

Supplementary figure 8

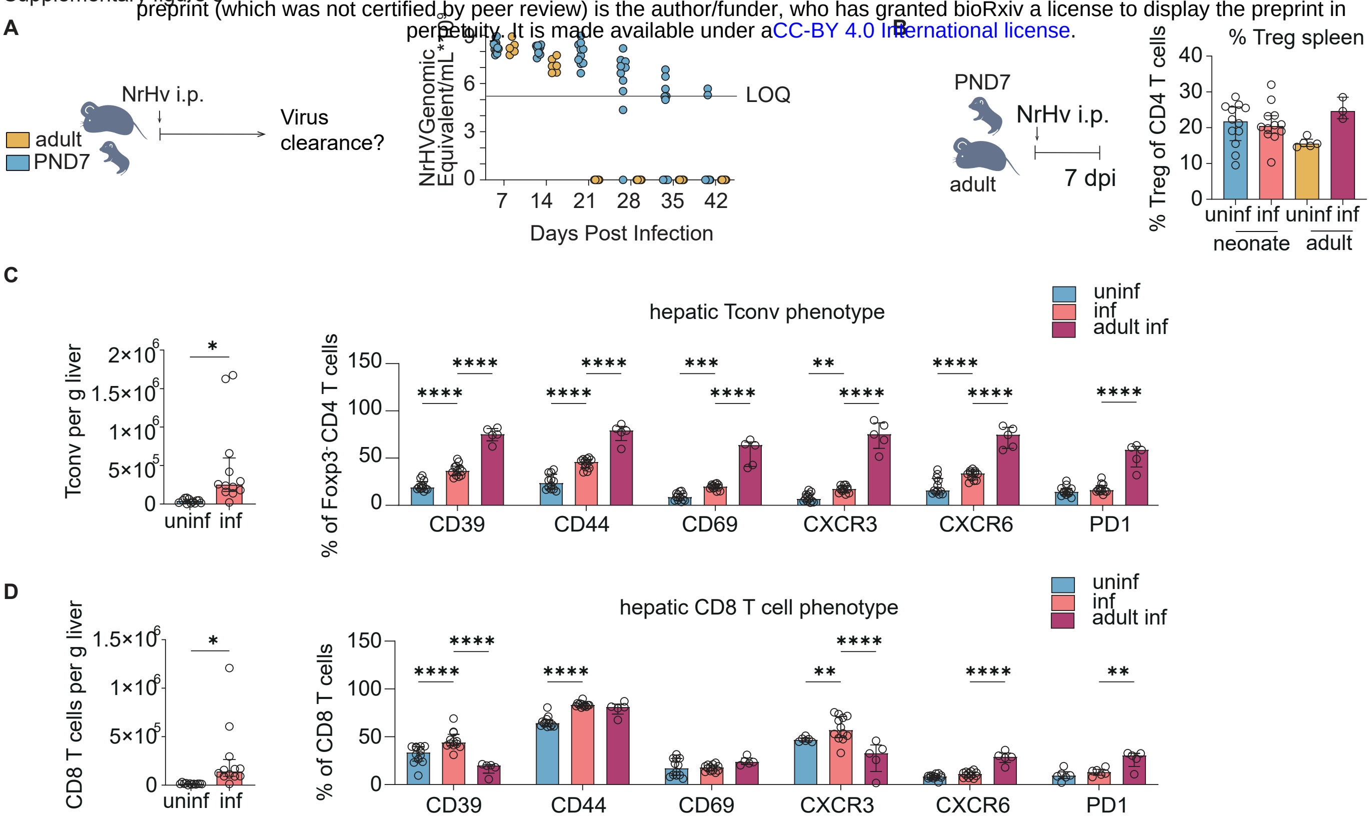

Supplementary figure 8: Extended data for analysis of NrHV infected mice

(A) Experimental design of neonatal NrHV infection. Viremia levels in neonatally or adult NrHV infected mice. 5-10 mice from 1 adult and 2 independent neonatal experiments.

(B) Splenic Treg in NrHV-infected and uninfected neonatal and adult mice. Flow cytometry data of 11-12 neonatal mice from 2 pooled independent experiments and 3-5 adult mice from 1 experiment. One Way ANOVA + Tukey`s multiple comparison test.

(C) Hepatic Tconv density and phenotypic markers in neonatal uninfected, neonatal infected and adult infected mice 7 days post-infection measured by flow cytometry. 12 neonatal mice per group from 2 independent experiments and 5 adult mice per group from 1 experiment. Two Way ANOVA with simple effects calculation between the neonatal infected group and the two other groups + multiple comparison correction via Holm-Sidak test.

(D) Hepatic CD8 T cell number and phenotypic markers in neonatal uninfected, neonatal infected and adult infected mice 7 days post-infection measured by flow cytometry. 12 neonatal mice per group from 2 independent experiments and 5 adult mice per group from 1 experiment. Two Way ANOVA with simple effects calculation between the neonatal infected group and the two other groups + multiple comparison correction via Holm-Sidak test.

bioRxiv preprint doi: https://doi.org/10.64898/2026.01.13.698576; this version posted January 13, 2026. The copyright holder for this preprint (which was not certified by peer review) is the author/funder, who has granted bioRxiv a license to display the preprint in perpetuity. It is made available under aCC-BY 4.0 International license.

Supplementary figure 9

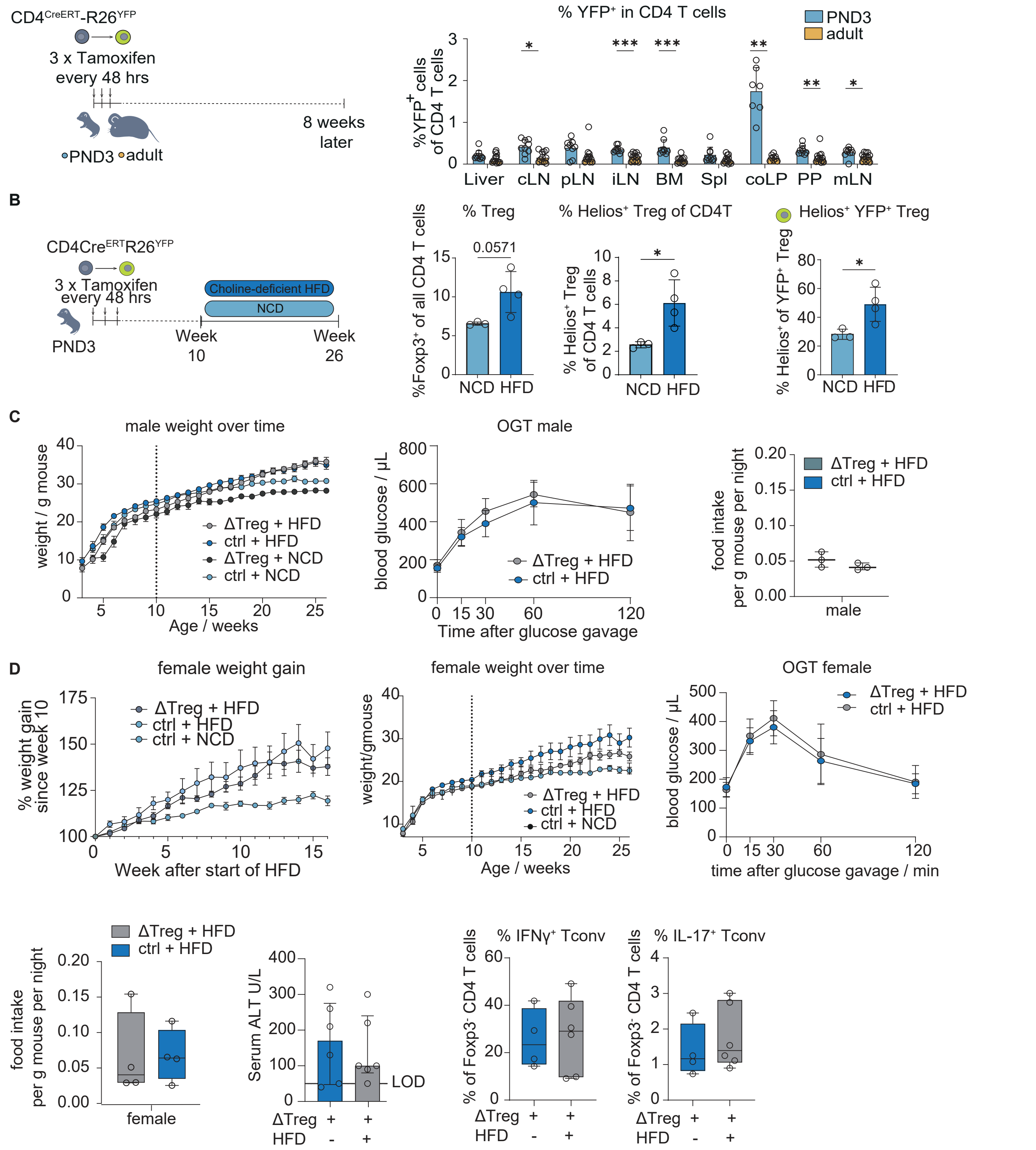

Supplementary figure 9: Timestamping and choline-deficient HFD experiments.

(A) Experimental design and % YFP⁺ of CD4 T cells in different organs 8 weeks after timestamping. Multiple Mann Whitney U tests.

(B) Experimental design of neonatal timestamping in combination with HFD. 1 representative of 2 experiments with littermates 3-4 mice per diet group. Unpaired t-tests.

(C) Extended data for male mice on HFD after neonatal Treg depletion. Body weight over the course of the experiment, oral glucose tolerance test. Food intake per g body weight per night in the last week of HFD (3 mice per group pooled from 2 independent experiments).

(D) Data for female mice on HFD after neonatal Treg depletion. Weight gain since start of HFD, body weight over the course of the experiment, oral glucose tolerance test. 7-8 mice per group from 6 independent experiments. Food intake per g body weight per night in the last week of HFD ((3 mice per group pooled from 2 independent experiments). 7 Serum ALT levels and flow cytometry measurement of IFNg and IL-17 producers in Tconvs after restimulation with PMA/Ionomycin. 4-6 per group from 4 independent experiments using only litters with both neoTreg and ctrl mice. Statistical analysis performed as was done for the male mice in the corresponding main figure.
